# Phylotranscriptomic Insights into the Diversification of Endothermic *Thunnus* Tunas

**DOI:** 10.1093/molbev/msy198

**Published:** 2018-10-26

**Authors:** Adam G Ciezarek, Owen G Osborne, Oliver N Shipley, Edward J Brooks, Sean R Tracey, Jaime D McAllister, Luke D Gardner, Michael J E Sternberg, Barbara Block, Vincent Savolainen

**Affiliations:** 1 Department of Life Sciences, Silwood Park Campus, Imperial College London, Ascot, United Kingdom; 2 Shark Research and Conservation Program, The Cape Eleuthera Institute, Rock Sound, Eleuthera, The Bahamas; 3 School of Marine and Atmospheric Science, Stony Brook University, Stony Brook, NY; 4 Institute for Marine and Antarctic Studies, University of Tasmania, Hobart, TAS, Australia; 5 Department of Biology, Hopkins Marine Station, Stanford University, Pacific Grove, CA; 6 Centre for Integrative Systems Biology and Bioinformatics, Department of Life Sciences, Imperial College London, Kensington, London, United Kingdom

**Keywords:** endothermy, thermogenesis, phylogenomics, RNA-sequencing, transcriptomics, introgression, positive selection, mitochondrial–nuclear discordance

## Abstract

Birds, mammals, and certain fishes, including tunas, opahs and lamnid sharks, are endothermic, conserving internally generated, metabolic heat to maintain body or tissue temperatures above that of the environment. Bluefin tunas are commercially important fishes worldwide, and some populations are threatened. They are renowned for their endothermy, maintaining elevated temperatures of the oxidative locomotor muscle, viscera, brain and eyes, and occupying cold, productive high-latitude waters. Less cold-tolerant tunas, such as yellowfin tuna, by contrast, remain in warm-temperate to tropical waters year-round, reproducing more rapidly than most temperate bluefin tuna populations, providing resiliency in the face of large-scale industrial fisheries. Despite the importance of these traits to not only fisheries but also habitat utilization and responses to climate change, little is known of the genetic processes underlying the diversification of tunas. In collecting and analyzing sequence data across 29,556 genes, we found that parallel selection on standing genetic variation is associated with the evolution of endothermy in bluefin tunas. This includes two shared substitutions in genes encoding glycerol-3 phosphate dehydrogenase, an enzyme that contributes to thermogenesis in bumblebees and mammals, as well as four genes involved in the Krebs cycle, oxidative phosphorylation, β-oxidation, and superoxide removal. Using phylogenetic techniques, we further illustrate that the eight *Thunnus* species are genetically distinct, but found evidence of mitochondrial genome introgression across two species. Phylogeny-based metrics highlight conservation needs for some of these species.

## Introduction

The *Thunnus* tuna clade consists of some of the most commercially important fish species in the world. The genus includes the three iconic bluefin species, which have all in recent times undergone precipitous population declines, some now recovering with careful management plans, but all remain the target of fisheries owing to their high commercial value (Matsuda et al. 1998; Safina and Klinger 2008; MacKenzie et al. 2009; ISC 2016). By contrast, other *Thunnus* species sustain huge global fishery yields; with the yellowfin tuna, *Thunnus albacares*, in particular comprising the seventh highest global landings of all fish species in 2014 (FAO 2016). Although the eight *Thunnus* species are thought to have diverged rapidly (Miya et al. 2013; Santini et al. 2013; Díaz-Arce et al. 2016), considerable ecological and physiological variability exists within the clade (Bernal et al. 2017).

All tuna species (also including the genera *Euthynnus*, *Auxis*, *Katsuwonus*, and *Allothunnus*) are regionally endothermic (supplementary fig. S1, Supplementary Material online). Unlike other teleosts, much of their aerobic red muscle is located near the center of the body. The evolution of vascular countercurrent heat exchangers has enabled the conservation of heat generated by metabolism and contraction in this muscle, allowing the maintenance of elevated tissue temperatures (Carey and Teal 1966; Block and Finnerty 1994; Sepulveda et al. 2008). The *Thunnus* tunas are particularly notable among the tunas as they have diversified rapidly and show a wide degree of variability in their distributions and thermal tolerances. Taxonomists initially split tunas of the genus *Thunnus* into two subgenera based on morphological characters: the tropical *Neothunnus* (including yellowfin, blackfin [*T. atlanticus*], and longtail [*T. tonggol*]) and the more high-latitude and cold-tolerant *Thunnus* (including Atlantic bluefin [*T. thynnus*], Pacific bluefin [*T. orientalis*], southern bluefin [*T. maccoyii*], bigeye [*T. obesus*], and albacore tuna [*T. alalunga*]; Gibbs and Collette 1967). These cold-tolerant *Thunnus* species, unlike the three species of the *Neothunnus* subgenus, have additional heat-exchangers around their viscera, enabling retention of heat generated during digestion and in some cases the brain and eye regions (Linthicum and Carey 1972). Increases of visceral temperature postdigestion that may increase speed of digestion and result in a larger thermal excess in cooler high latitude (Whitlock et al. 2015). The albacore tuna also extends its range into high-latitude waters, preferring waters where the sea surface temperatures are as low as 14 °C (Arrizabalaga et al. 2015). The bigeye tuna occupies tropical and subtropical waters, but spends considerable time diving to deep mesopelagic resources, encountering cool waters, before returning to the surface to warm up (Holland and Sibert 1994; Schaefer and Fuller 2010). Although it was placed in the *Thunnus* subgenus, it shares characteristics with the tropical *Neothunnus* and was considered an intermediate between the two (Gibbs and Collette 1967). Electronic tagging has shown that the three bluefin tuna species are especially cold-tolerant, feeding as large adults in high latitudes in subpolar waters where sea temperatures can be as low as 9 °C at the surface and 0–2 °C at depth (Bestley et al. 2009; Block et al. 2011; Arrizabalaga et al. 2015; Wilson et al. 2015).

The capacity of bluefin tunas to spend prolonged periods of time in cool subpolar and temperate waters has been hypothesized to be associated with increases in cardiac capacity and aerobic metabolism. Among *Thunnus* species with measurements to date, bluefin tunas have been shown to have elevated cardiac capacities particularly in excitation–contraction coupling (Landeira-Fernandez et al. 2004) and an increased capacity to maintain cardiac ion channels conductance at low temperatures (Galli et al. 2011). The deep red muscle of yellowfin tuna is known to be specialized to operate at high temperatures, being very sensitive to temperature decrease (Altringham and Block 1997). This suggests that even at low ambient water temperatures, elevated temperatures must be maintained in deep muscle to maintain near-optimal function. Similar physiological studies have not been carried out in any bluefin tuna species, and so it is not known whether there deep red muscle is equally thermally sensitive. However, large Atlantic bluefin tuna are known to maintain stable elevated temperatures in deep muscle at ambient water temperatures of 9–17 °C (Stevens et al. 2000). This has led authors to suggest that the capacity of bluefin tunas to occupy high latitudes with low sea surface temperature is associated with increased endothermic capacity (Blank, Farwell, et al. 2007), as higher thermal gradients between deep muscle and the ambient water must be maintained for prolonged periods. At low temperatures below their thermal optimum, the metabolic rate of Pacific bluefin tuna increases. This is atypical of ectothermic fish, where metabolic rate would decrease with temperature, but typical of endothermic animals (Blank, Morrissette, et al. 2007). This suggests that upregulation of aerobic metabolism may be associated with thermoregulation and endothermy in these species.

Another aspect in which the *Thunnus* vary is in their reproductive biology. The three species of the subgenus *Neothunnus*, alongside the bigeye, remain in warm-temperate to tropical waters year-round. These four species are thought to spawn throughout much of the year. By contrast, the albacore and bluefin tunas have more restricted spawning seasons, as they spend much of the year feeding in productive higher-latitude waters, returning to subtropical or tropical seas to spawn for short durations (Schaefer 2001; Muhling et al. 2017). Higher fecundities and generally faster generation times of tropical tunas (Juan-Jordá et al. 2013) have counterbalanced enormous fishing pressure (Juan-Jordá et al. 2015), although bigeye and yellowfin populations are, in some regions, decreasing in size (table 2). Life-history traits are therefore critical to the survival of tunas in modern oceans where humans have increased predation pressure (Kroodsma et al. 2018). Despite the relevance to high-stakes fisheries, little is known of the evolutionary processes to have driven this physiological and ecological diversification of the *Thunnus* clade.

**Table 2.** Fishing Pressure, Conservation Status, and Calculated Evolutionary Distinctness and EDGE Scores.

Common Name	Species Name	2015 Global Fisheries Yield (tonnes, FAO)	IUCN Red List Status (GE score for EDGE calculation; IUCN 2017)	Global Spawning Stock Biomass Change Over Past Three Generations (IUCN 2017)	Evolutionary Distinctness (ED)	EDGE Score
Albacore tuna	*Thunnus alalunga*	223,013	Near-threatened (1)	−37%	12.2	3.3
Yellowfin tuna	*Thunnus albacares*	1,359,192	Near-threatened (1)	−33%	9.0	3.0
Blackfin tuna	*Thunnus atlanticus*	1,420	Least concern (0)	Stable	8.8	2.3
Southern bluefin tuna	*Thunnus maccoyii*	21,837	Critically endangered (4)	−85%	9.6	5.1
Bigeye tuna	*Thunnus obesus*	417,336	Vulnerable (2)	−42%	9.0	3.7
Pacific bluefin tuna	*Thunnus orientalis*	35,524	Vulnerable (2)	−19% to 33%	8.1	3.6
Atlantic bluefin tuna	*Thunnus thynnus*	23,811	Endangered (3)	−51%	8.1	4.3
Longtail tuna	*Thunnus tonggol*	201,894	Data deficient (—)	Unknown	8.8	—

Estimates of phylogenetic relationships using partial genomic data (Díaz-Arce et al. 2016) and mitochondrial sequence data (Chow and Kishino 1995; Bayona-Vásquez et al. 2017) have suggested that the three bluefin tuna species are paraphyletic. In the partial genomic data phylogeny, the southern bluefin is sister to the warm-water tuna clade (supplementary fig. S2, Supplementary Material online). By contrast, in the mitochondrial genome phylogenies, Atlantic and southern bluefins are sister species, but Pacific bluefin is sister taxa to the albacore (supplementary fig. S2, Supplementary Material online). Prior to the advent of mitochondrial phylogenetics, Pacific and Atlantic bluefin tunas were considered a single species (Chow and Kishino 1995; Collette et al. 2001). These northern bluefins are thought to be only weakly differentiated in the nuclear genome (Chow et al. 2006; Díaz-Arce et al. 2016). This mitochondrial–nuclear discordance has been used to hypothesize introgression between albacore and Pacific bluefin tuna. However, this may also be driven by incomplete lineage sorting (ILS; Toews and Brelsford 2012), which has not been tested. Rapid radiations are generally associated with a high degree of gene-tree discordance, where different genes have conflicting topologies (Pamilo and Nei 1988). This may be a result of both ancestral hybridization events and failure of ancestral genetic variation to sort in-between speciation events, resulting in ILS (Maddison 1997). Given the rapid divergence and large population sizes of *Thunnus* tuna, ILS is likely to have generated significant gene-tree discordance. This may have misled phylogenies of the *Thunnus* tuna to date, as “supermatrix” techniques they utilized may be inaccurate when genealogical discordance is high (Kubatko et al. 2007; Pirie 2015; Díaz-Arce et al. 2016; Mendes and Hahn 2017). Genealogical discordance may also explain why the evolution of traits in a rapid radiation may not correlate with monophyletic relationships in the species tree. This may be explained by processes such as parallel selection on standing variation or introgression (Hahn and Nakhleh 2016; Pease et al. 2016).

Here, we used an RNA-seq data set consisting of multiple individuals of each *Thunnus* species to explore the evolutionary processes underlying their diversification. The first aim of our study was to clarify phylogenetic relationship among the *Thunnus* species. The second aim was to assess how hybridization, selection on standing variation, and de novo mutation have shaped the *Thunnus* radiation. We find that de novo mutation has played a role in the evolution of the tropical group and that selection on standing variation has driven the phenotypic divergence of cold tolerance in bluefin tunas. This includes bluefin-specific variants in genes associated with key metabolic and thermogenic functions.

## Results and Discussion

To elucidate the evolutionary history of *Thunnus*, and to learn more about the evolution of endothermy, specifically in the bluefin tuna and visceral endotherm groups, we collected RNA-sequence data for 25 individual tunas, supplementing NCBI Short Read Archive data to reach a total of 46 individuals (supplementary table S1, Supplementary Material online). This transcriptomic data set included at least two individuals from each of the eight *Thunnus* species, plus the skipjack tuna, *Katsuwonus pelamis*, as an outgroup. We note that there was some variation in tissue type used among the 46 individuals, which may have reduced coverage of tissue-specific genes. We first generated a merged de novo assembly based on 102 unique assemblies from skeletal muscle (red and white muscle) and heart tissue (compact ventricle, spongy ventricle, and atrium) of three individual Pacific bluefin tuna. Multiple tissue types were used to provide a more complete reference assembly. The merged assembly comprised 48,648 transcripts, corresponding to 29,556 genes. This merged assembly was more complete and had less redundancy than any of the individual assemblies (complete sequences for 89.1% of a bony fish single-copy ortholog set [see Materials and Methods]; supplementary table S2 and fig. S3, Supplementary Material online). Therefore, sequence data from each of the 46 individuals were mapped and genotyped against this merged reference transcriptome.

### Introgression Evident in Mitochondrial, but Not Nuclear Genomes of Tunas

**Fig. 1.**
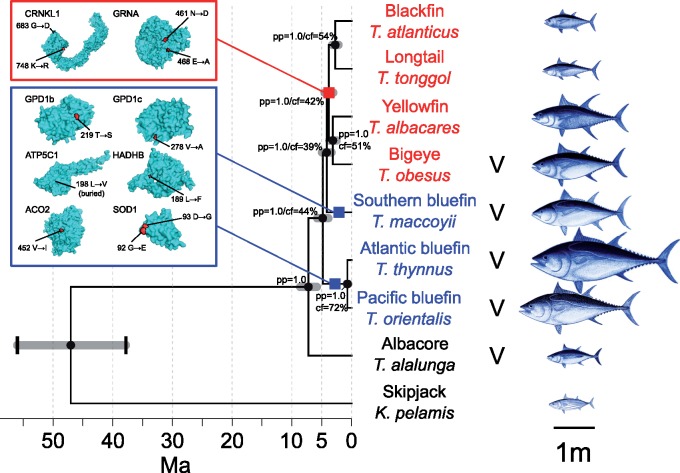
Fossil-dated phylogeny of tunas and parallel selection in bluefin species. 3D surface protein structures for genes with shared nonsynonymous mutations in bluefin tunas and a function relating to aerobic metabolism are given in the blue box, with the two branches where parallel selection on these variants occurred highlighted with blue squares, with bluefin species highlighted in blue. 3D protein structures inferred for genes under lineage-specific selection in the warm-water group are given in the red box. The branch these changes correspond to is indicated with a red square, with warm-water species highlighted in red. Species with visceral endothermy are indicated with a “V.” Amino acid changes and positions on the zebrafish reference genome (see table 1) are given, and their location on each protein model is highlighted in red. Species illustrations are from the FAO and wikimedia, rescaled according to the maximum length of each species, taken from Juan-Jordá et al. (2013). Gray error bars show 95% confidence intervals of divergence-date estimates. Black brackets on root node indicate minimum and maximum fossil calibration. Node labels are Bayesian posterior probability (pp), followed by concordance factors (cf) for the primary quartet inferred by ASTRAL; values lower that 100% indicate increasing gene-tree discordance, which in this case are within expectation from ILS.

**Fig. 2.**
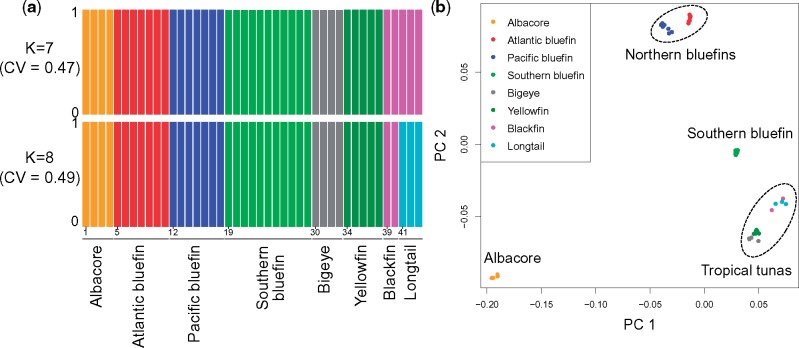
Genetic structure in tuna. (*a*) ADMIXTURE plot showing the estimated membership coefficients for each individual (labeled from 1 to 43 at the start of each species group), in each cluster. Each individual is represented by a single vertical bar, which is partitioned into *K* colored segments. Here the best scoring values were *K = *7 (top figure), and then *K *=* *8 (where all individuals cluster per species, lower plot), according to ADMIXTURE CV. (*b*) MDS for independent SNPs across the *Thunnus* phylogenetic tree, highlighting four groups. These analyses found no evidence of admixture between species (see text for details).

Using either coalescence or concatenated-gene (supermatrix) phylogenetic analyses, we inferred the same phylogeny. This was consistent using coalescent trees based on either gene trees with poorly supported branches collapsed into hard polytomies or not, and with supermatrix trees based on either 4-fold degenerate sites or full transcripts, with all nodes in the trees supported by a posterior probability of 1 or bootstrap support of 100% (supplementary fig. S4, Supplementary Material online). Importantly, this phylogenetic analysis demonstrates that both the bluefin tunas and visceral endotherms are paraphyletic, as was suggested by partial-genomic data (Díaz-Arce et al. 2016). All individuals within each species formed monophyletic groups (supplementary fig. S5, Supplementary Material online), with Atlantic and Pacific bluefin tunas being segregated as distinct taxa. The Atlantic and Pacific bluefin tuna distinction is further supported by a Bayes Factor Delimitation model (posterior probability = 0.999). Dating the *Thunnus* phylogeny using fossil calibration shows that this lineage has radiated rapidly within the last 6–10 My (fig. 1). Notably, a high level of gene-tree versus species-tree discordance is observed, as indicated by quartet concordance factors <50% at internal nodes (fig. 1). We calculated that this discordance did not deviate from expectations under ILS (*P *=* *0.2), which argues against ancestral hybridization events evident in the nuclear genome (supplementary fig. S6, Supplementary Material online). This idea is further supported by hierarchical clustering and multidimensional scaling (MDS) analyses, which both indicated no admixture between species (fig. 2). Only longtail and blackfin tunas (two species with lowest sample sizes) fail to segregate in the best-fitting hierarchical clustering model (seven ancestral populations, cross-validation [CV] error = 0.47; supplementary fig. S7, Supplementary Material online), although they do so in the eight ancestral population model (CV error = 0.49; fig. 2) and in the phylogenetic trees (supplementary fig. S6, Supplementary Material online).

We did, however, find significant mitochondrial–nuclear discordance (supplementary fig. S8, Supplementary Material online). In the mitochondrial phylogenetic tree, as in other mitochondrial studies (Chow and Kishino 1995; Qiu et al. 2014; Bayona-Vásquez et al. 2017), the Pacific bluefin and the albacore are clustered. In the nuclear-based tree the Pacific bluefin is sister to the Atlantic bluefin, more distantly related to the albacore (fig. 1). This discordance has been used as evidence of introgression between Pacific bluefin and the albacore (Chow and Kishino 1995; Bayona-Vásquez et al. 2017). By simulating gene trees, we found that the sister relationship of Pacific bluefin and albacore tunas is unlikely due to ILS alone (*P *=* *0.02). This shows that this mitochondrial–nuclear discordance is indeed likely due to introgression.

Taken together, we find that *Thunnus* tuna show evidence of introgression in the mitochondrial, but not nuclear genomes. This has also been observed in a wide range of taxa (Zieliński et al. 2013; Pons et al. 2014; Good et al. 2015), and is likely when selection has favored these mitochondrial variants and background selection and recombination has removed the introgressed nuclear variants (Bonnet et al. 2017; Sloan et al. 2017). However, this does little to explain the evolution of endothermy in bluefin tunas. Instead, much of the nuclear gene-tree discordance is likely due to standing variation from the ancestral populations being retained during a rapid radiation, as not all the variation has had time to be fixed between species splits (Pamilo and Nei 1988). We note that power to detect hybridization events is reduced as time since hybridization increases, particularly when speciation events are rapid (Folk et al. 2018). We therefore cannot conclusively rule out hybridization playing a greater role in the *Thunnus* radiation.

### Parallel Selection on Standing Genetic Variation in Bluefin Tuna

To test whether parallel selection on this ancestral genetic variation underlies the evolution of endothermy in bluefin tunas or visceral endotherms, we used a phylogenetic genome-wide association test (PhyloGWAS; Pease et al. 2016). We found that there was an excess of genes with nonsynonymous mutations shared by the three bluefin tuna species compared with expectations due to ILS alone (*P *<* *0.0006). By contrast, there was no excess of shared nonsynonymous mutations between the visceral endotherms (*P *=* *0.14). We found parallel selection on standing genetic variation in 96 genes in the bluefin tunas (supplementary table S3, Supplementary Material online). Functional gene ontology (GO) enrichment analysis indicated enrichment in several terms relating to glycerol-3-phosphate dehydrogenase (GPDH) activity (supplementary table S4, Supplementary Material online). Furthermore, bluefin-specific nonsynonymous mutations were found in genes that are functionally related to aerobic metabolism (Blank, Farwell, et al. 2007; Wiens et al. 2017) and relevant to the evolution of endothermy (table 1; Matsuda et al. 1993; Mattiazzi et al. 2002; Lushchak et al. 2014; Naiki et al. 2014). These genes are all characterized by one or two bluefin group-specific nonsynonymous substitutions (fig. 1). This is consistent with previous studies that have found that the vast majority of genes with variants fixed by selection on standing variation are characterized by one or just a few mutations (Pease et al. 2016; Wu et al. 2017). Single mutations can, however, have strong effects on phenotype (Mattiazzi et al. 2002; Fox et al. 2017).

**Table 1.** Candidate Genes Underlying the Evolution of Endothermy in Bluefin Tunas (see text for details).

Gene Name	Gene Abbreviation	Uniprot Reference Sequence and Site of Mutation	Consurf Amino Acid Site Phylogenetic Conservation Score (1 = highly variable, 9 = highly conserved)	Change in Protein Stability (pseudofolding-free energy ΔΔ*G*)	Significant Change in Electrostatic Potential? (nonparametric Wilcoxon signed-rank test)	Putative Function
Glycerol-3-phosphate dehydrogenase 1b	*GPD1b*	F1QGK0_DANRE: 219	3	−0.8	Increase (*Z*-score 3.37)	Transfers cytosolic NADH, produced by glycolysis, to mitochondrial glycerol-3-phosphate dehydrogenase as NAD+, which then feeds oxidative phosphorylation (McDonald et al. 2017)
Glycerol-3-phosphate dehydrogenase 1c	*GPD1c*	Q7T3H5_DANRE: 278	6	−0.9	No (*Z*-score 1.6)	As with *GPD1b*
Aconitase 2	*ACO2*	F8W4M7_DANRE: 452	2	−0.3	Decrease (*Z*-score −2.1)	Mitochondrial aconitase isoform. Controls cellular ATP production by regulating intermediate flux in the Krebs cycle (Lushchak et al. 2014)
ATP synthase, H+ transporting, mitochondrial F1 complex, gamma polypeptide 1	*ATP5C1*	Q6P959_DANRE: 198	4	−2.1	No (*Z*-score 0.8)	Encodes gamma subunit of mitochondrial ATP synthase. This catalyzes ATP synthesis during oxidative phosphorylation (Matsuda et al. 1993)
Hydroxyacyl-CoA dehydrogenase/3-ketoacyl-CoA thiolase/enoyl-CoA hydratase (trifunctional protein), beta subunit	*HADHB*	Q7ZTH6_DANRE: 189	1	−0.6	Decrease (*Z*-score −5.1)	Subunit of mitochondrial trifunctional protein, which catalyzes the last three steps of mitochondrial β-oxidation of long-chain fatty acids. This in turn feeds the krebs cycle and aerobic metabolism (Naiki et al. 2014)
Superoxide dismutase 1, soluble	*SOD1*	SODC_DANRE: 92 and 93	Site 92: 1 Site 93: 2	Site 92: +0.4 Site 93: −1.1	Site 92: No (*Z*-score 0.7) Site 93: Increase (*Z*-score 4.4)	Destroys toxic free radicals, the majority of which are produced by mitochondria (Mattiazzi et al. 2002)

Note.—Amino acid changes are provided in figure 1.

To understand the evolution of endothermy it is critical to elucidate how incremental increases in metabolic rate, oxidative pathways, and thermogenesis occur in any lineage. Studies to date have suggested metabolic rates may be higher in bluefin tuna species; Pacific bluefin tuna have higher metabolic rates than yellowfin tuna, acclimated at the same temperatures and caught in the same California current waters. These fish were trained to swim in a flume at the same swimming speed and were of similar size (Blank, Farwell, et al. 2007). Measurements of southern bluefin tuna metabolic rates have also exceeded those from skipjack, albacore, kawakawa tuna (*Euthynnus affinis*), and yellowfin (Fitzgibbon et al. 2008). However, accurately measuring metabolic rates of large, active pelagic fish is difficult, and comparing between studies is difficult due to experimental variation which may substantially impact results.

Here we found bluefin tuna-specific mutations in six genes associated with aerobic capacity (table 1), supporting the view of unique adaptations in aerobic metabolism in the bluefin tunas. This includes isoforms of *GPD1* (*GPD1b* and *GPD1c*), which works in concert with mitochondrial *GPD2* to form the glycerol-3-phosphate (G3P) shuttle. This pathway uses the NADH synthesized during glycolysis to contribute electrons to the oxidative phosphorylation pathway in the mitochondria, fueling ATP synthesis. ATP synthesis by G3P-mediated respiration is inefficient, as only two ATP molecules are synthesized per NADH molecule, instead of the three ATP derived from NADH formed inside the mitochondria. However, it sustains a high rate of oxidative phosphorylation (Gong et al. 1998). This metabolic inefficiency, coupled with the high oxidative phosphorylation rates, produces heat elevating tissue temperatures locally. This pathway has been found to be important for thermogenesis in mammals and bumble bee flight muscles (Gong et al. 1998; Masson et al. 2017). GPDH also plays an important role in lipid metabolism (Hao et al. 2015), which these mutations also may relate to. Selection for aerobic metabolism in bluefin tunas is further implicated by mutations in key oxidative phosphorylation genes (*ATP5C1*; Matsuda et al. 1993), Krebs cycle (*ACO2*; Lushchak et al. 2014) and β-oxidation (*HADHB*; Naiki et al. 2014) genes, as well as in *SOD1.* This latter gene codes for the enzyme that removes toxic reactive oxygen species (ROS) produced during aerobic respiration (Mattiazzi et al. 2002). Recent measurements on isolated mitochondria of Pacific bluefin tuna indicate that they produce ROS at a similar rate to ectothermic fish species at a similar temperature (Wiens et al. 2017). However, this rate is temperature dependent, meaning that the elevated body temperature in bluefin tuna tissues will increase metabolism and ROS production and increase the risk of mitochondrial damage (Murphy 2009). Notably, the amino acid substitutions in *SOD1* in bluefin tunas (fig. 1) are both adjacent to a well-characterized mutation site in mice. G93A transgenic mice show significant defects in mitochondrial function due to increased oxidative damage (Mattiazzi et al. 2002). The proximity of the bluefin substitutions to this site suggests that they possibly relate to reducing oxidative damage, which would be exacerbated by elevated metabolic rates associated with selection for endothermy.

Using 3D-structure models predicted using Phyre2 (Kelley et al. 2015) for each of these proteins, we identified that all nonsynonymous mutations fell at amino acid sites on the surface of the protein, except for that in *ATP5C1* (fig. 1). None of these mutations is particularly conserved across other organisms (ConSurf score 1–6). However, we identified that these amino acid changes significantly alter either protein electrostatic potential or stability (table 1), which likely indicate functional roles associated with these mutations. Overall, our analyses indicate that parallel selection on genetic variants relating to both aerobic metabolism pathways and oxygen utilization has contributed to the unique phenotype of bluefin tunas. Experimental validation of these candidate genes, alongside detailed analysis of the G3P-shuttle in the context of isolated mitochondrial function and oxidative phosphorylation in tuna is now necessary. These experiments could determine whether selection for G3P-mediated respiration provides novel pathways for heat production in bluefin tunas, as in bees and mammals.

### Positive Selection in Warm-Water Tunas

Our transcriptomic data indicate that warm-water and tropical tunas (bigeye, yellowfin, longtail, and blackfin) form a clade (fig. 1). These fish tend to occupy tropical and warm-temperate waters for longer durations than bluefin tunas throughout the year (Block et al. 2011), with most populations staying in warm-temperate to tropical waters year-round, and in general mature earlier (Juan-Jordá et al. 2013). In these warm-water tunas, we detected selection on de novo lineage-specific mutations in two genes, both with possible functions linked to growth and embryogenesis but not endothermy: crooked neck pre-MRNA splicing factor 1 (*CRNKL1*, CodeML branch-site test *P *=* *0.0007) and granulin a (*GRNA*, *P *=* *0.004; Bateman and Bennett 1998; Chung et al. 2002). As with the bluefin genes, the amino acid changes were at the surface (fig. 1), in variable amino acid sites (ConSurf score 1–6), but with significant impacts on the overall protein electrostatic potential. The substitution in *GRNA* at zebrafish amino acid-site 461 decreased electrostatic potential (nonparametric Wilcoxon signed-rank test *Z*-score = −5.8), whereas that at 468 increased it (*Z*-score = 5.9). The substitution at *CRNKL1* site 683 decreased electrostatic potential (*Z*-score = −4), whereas that at site 748 did not (Z-score = 0.3). None of these substitutions strongly influenced protein stability (pseudo ΔΔG −0.09 to 0.32). Importantly, many more genes specifically associated with reproduction and maturation may not have been expressed in our muscle samples.

### Bluefin Tuna Species Are Evolutionarily Distinct and Globally Endangered

Finally, combining red list status from the World Conservation Union (IUCN) and branch lengths in our phylogenetic trees, we calculated EDGE scores for each species (i.e., evolutionary distinctness and globally endangered status [Isaac et al. 2007], table 2). This score is a popular metric in conservation biology, as it identifies those threatened species that deserve particular attention because of their unique evolutionary history. Relative to other tunas, we found the highest scores in southern bluefin (5.1) and Atlantic bluefin (4.3). We also highlight the importance of gathering data for the longtail tuna, which is currently classified as “data-deficient,” but has a substantial global fishery yield of 201,894 tonnes in 2015 (table 2). Longtail tuna may be increasingly targeted directly and as bycatch, as the populations of other species with large fisheries are decreasing or are quota limited.

Tunas are unusual among bony fish for their evolution of endothermy (Block et al. 1993). Our analyses shed light on the phylogeny and genetic basis of the evolution of endothermy in tunas. We found bluefin-specific nonsynonymous mutations in six key aerobic metabolism genes, supporting the hypothesis that adaptations in aerobic metabolism are key to the endothermy of these species, and their ability to expand their niche into high latitudes. Further studies may wish to consider whether there has been any genetic convergence with the slender tuna, *Allothunnus fallai*, whose phylogenetic placement is currently unclear (Sepulveda et al. 2008; Santini et al. 2013). Because we have shown a high degree of gene-tree discordance among the *Thunnus* species, indicating a high degree of ancestral variation, trait evolution should not be considered to show a stepwise progression across the *Thunnus* phylogeny. This will influence how we analyze the diversification of further important traits as increasing information becomes available. For example, hypoxia tolerance varies considerably among the five *Thunnus* species for which it has been measured, inconsistently with the current species tree (Bernal et al. 2017). This trait will have strong impacts on future tuna distributions as deoxygenation of the ocean will increase under global warming scenarios (Breitburg et al. 2018). The rapid availability of fully sequenced genomic resources particularly from bluefin tunas will improve our capacity to study these unique fish.

## Materials and Methods

### Sample Collection and RNA Sequencing

Samples were collected from multiple individuals of all eight *Thunnus* species along with the Skipjack tuna, *Katsuwonus pelamis*, which was used as an outgroup. Short-read sequence data downloaded from the NCBI Short Read Archive for 19 individuals were supplemented with samples collected from fish markets (the United Kingdom and the Netherlands) and from the wild (Bahamas, southern Australia, and California; supplementary table S1, Supplementary Material online) by the Tuna Research and Conservation Center, University of Tasmania, and Cape Eleuthera Institute. Tissue samples stored in RNALater (Thermo Fisher Scientific, Waltham, MA) were sent to BGI Tech Solutions, Hong Kong. There, RNA was extracted using TRIzol (Invitrogen, Carlsbad, CA). Using the TruSeq RNA Library Preparation Kit (v2), cDNA libraries were produced. These were then sequenced using the Illumina HiSeq 4000 (Illumina Inc, San Diego, CA) with 100-bp paired-end reads. Raw sequencing reads have been deposited to NCBI GenBank under BioProject PRJNA495053.

### Read Processing and Reference Transcriptome

Initial quality control was carried out by BGI Tech Solutions, with low-quality reads (average phred < 20), primer and adapter sequences trimmed. Upon retrieval, sequencing errors in these reads were corrected using Rcorrector (V1.0.2; Song and Florea 2015), then further trimmed for low-quality bases and adaptor sequences (phred < 2, following Macmanes [2014]), using Trim Galore! (v0.4.0; http://www.bioinformatics.babraham.ac.uk/projects/trim_galore/; last accessed March 31, 2017). Reads for each of the three Pacific bluefin tuna individuals with multiple tissue types sequenced were normalized in silico to a depth of 100× using Trinity (v2.4.0; Grabherr et al. 2011). Separate assemblies were carried out for each of the three individuals, using multiple assembly softwares and *k-*mer length settings. For Trinity (v2.4.0), Bridger (v2014-12-01; Chang et al. 2015), and Binpacker (v1.0; Liu et al. 2016), *k-*mer values of 19, 25, and 32 were used. For SOAPdenovo-trans (v1.03; Xie et al. 2014), Velvet-OASES (Velvet v1.2.10, OASES v0.2.08; Zerbino and Birney 2008; Schulz et al. 2012), Trans-ABySS (v1.5.5; Robertson et al. 2010), IDBA-tran (v1.1.0; Peng et al. 2013), and Shannon (v0.0.2; Kannan et al. 2016), *k*-mer values of 21, 31, 41, 51, 61, and 71 were used. This resulted in 34 assemblies for each of the three Pacific bluefin tuna individuals (three each for Trinity, Bridger, and Binpacker; six each for SOAPdenovo-trans, Velvet-OASES, Trans-ABySS, and Shannon; one for IDBA-tran, which builds on each previous *k*-mer length assembly resulting in one final assembly), for 102 unique assemblies in total. Only transcripts of at least 300 bp were retained. Coding sequences (CDS) were inferred from these using TransDecoder (v3.0.1; Grabherr et al. 2011). CDS from all 102 assemblies were concatenated and clustered using CD-HIT-EST (Fu et al. 2012), with the settings -aL 0.005 -aS 1 -c 0.97 -d 0 -G 0 -M 0 (Cerveau and Jackson 2016). Contigs generated by multiple assembly softwares and *k-*mer settings are less likely to be artifacts (Cerveau and Jackson 2016; Durai and Schulz 2016). We therefore retained the longest CDS representative of clusters containing clusters corresponding to at least an average of two assembly softwares with two *k*-mer settings each per individual. The transcript corresponding to each of these CDS was then extracted to give the final merged assembly. Completeness of the merged assembly and individual assemblies were assessed using BUSCO (Benchmarking Universal Single-Copy Orthologs; Simão et al. 2015) and the Actinopterygii_odb9 database (supplementary table S2, Supplementary Material online). A transcript-to-gene map was constructed using CORSET (Davidson and Oshlack 2014) and the mappings of the three Pacific bluefin tuna used to construct the final reference transcriptome.

CDS from the transcriptome were annotated against a database of teleost species (*Astyanax mexicanus*, *Danio rerio*, *Gasterosteus aculeatus*, *Gadus morhua*, *Lepisosteus oculatus*, *Oreochromis nilotocus*, *Oryzias latipes*, *Poecilia formosa*, *Tetraodon nigroviridis*, *Takifugu rubripes*, and *Xiphophorus maculatus*); protein sequences were downloaded from the ENSEMBL database in June 2017 (Aken et al. 2017) using NCBI BlastX (v2.6.0; Altschul et al. 1990). GO terms were extracted for each using the “biomartr” R package (Drost and Paszkowski 2017). If ENSEMBL sequences were not annotated, their protein sequence was annotated by NCBI BlastP search against the NCBI nr (nonredundant) database.

### Read Alignment

Reads from all individuals were separately mapped against this reference transcriptome using STAR (v2.5.3a; Dobin et al. 2013) and the double-pass method, allowing for any number of hits, and scoring all hits with an equal best mapping score as primary. Reads were realigned around indels using GATK (v3.7; McKenna et al. 2010). Genotypes were then inferred using samtools and bcftools (v1.5; Li et al. 2009; Li 2011). Bases with a base quality <20 were filtered using *samtools mpileup*. Using *bcftools call* and *filter*, heterozygous sites with either allele represented by <2 reads were trimmed, as were sites with high-quality read depth <3, genotype or variant quality <20 and single nucleotide polymorphisms (SNPs) within 3 bp of an indel. Resultant vcf files were converted to multisample fasta files using vcf2fas (v17072015; https://github.com/brunonevado/vcf2fas. Last accessed June 16, 2017. Indels were coded as missing data. IUPAC (International Union of Pure and Applied Chemistry) ambiguity codes were used for heterozygous sites.

### Phylogenetic Reconstructions

Phylogenetic trees were reconstructed using both supermatrix and summary multispecies coalescent (MSC) tools. For the MSC tree, transcript trees were inferred for each transcript. These were first filtered to remove columns with <10% occupancy and sequences with >50% gaps (Sayyari et al. 2017). Transcripts that then had sequences from <4 species were discarded. Trees were then inferred for each using RAxML (v8.2.10; Stamatakis 2014), with 200 rapid bootstraps and the GTRGAMMA model of evolution. SH-like (Shimodaira–Hasegawa-like) node support values were subsequently calculated (Anisimova et al. 2011). Transcript trees were discarded if the three skipjack tuna individuals (where sequence data were present) were not monophyletic, to remove trees with unrealistic deep coalescences. Transcript trees with at least one node with SH-like support >10 were retained, with only the transcript with most nodes with SH-like support >10 per CORSET cluster retained, in order to ensure the independence of markers. The species tree was then inferred using ASTRAL (v5.5.6; Mirarab et al. 2014). Two runs were carried out with forced species monophyly: one with poorly supported nodes (SH-like <10) collapsed into hard polytomies, as recommended by Zhang et al. (2018), and another where they were not. Gene-tree concordance values of each primary split, calculated using ASTRAL, in the first of these trees are reported. These indicate the percentage of gene trees supporting the species–tree relationship for each branch within a tree. A further run was carried out with the hard polytomy data set without forced species monophyly in order to ensure each species formed monophyletic groups.

Supermatrix-based phylogenetic trees were also inferred. Fixed nucleotides for each species from all the transcripts used in the ASTRAL phylogeny were concatenated. A maximum-likelihood phylogeny was inferred using RAxML (v8.2.1) with 200 rapid bootstraps and the GTRGAMMA model of evolution. A Bayesian phylogenetic tree was also inferred using ExaBayes (v1.5; Aberer et al. 2014). Four runs of three coupled chains were carried out for 1 million generations (25% as burn-in). In order to ensure Monte Carlo Markov chains were run long enough for accurate estimation of posterior means, we ensured the effective sample size were >200 for all parameters, alongside a potential scale reduction factor of 1 in order to assess convergence between chains. To account for the potential effect of selection, a further Bayesian phylogenetic tree was also inferred for concatenated 4-fold degenerate sites from the transcript set.

To infer a mitochondrial phylogenetic tree, reads from all *Thunnus* individuals were mapped against a reference Pacific bluefin tuna mitochondrial genome (NCBI accession number: NC_008455), with *Katsuwonus* individuals mapped against a reference skipjack tuna mitochondrial genome (NC_005316). Reads were mapped using STAR as above except allowing a maximum of two hits per read. They were subsequently genotyped and converted to fasta using samtools and bcftools. This was as above, except using the bcftools call setting “–ploidy 1,” not using homozygous blocks. CDS for each of the 13 genes of the mitochondrial genome for each individual were extracted using the MITOS webserver (Bernt et al. 2013). These were aligned using MAFFT (v7.271; Katoh and Standley 2013) and concatenated. A phylogenetic tree was then inferred, using ExaBayes, as with the nuclear supermatrix tree. The species identity of all individuals was verified by NCBI BlastN search of these mitochondrial CDS against the NCBI nr database, in addition to species monophyly in mitochondrial (supplementary fig. S8, Supplementary Material online) and nuclear (supplementary fig. S6, Supplementary Material online) phylogenetic trees.

### Test for Species Delimitation

To test for species delimitation between the Atlantic and Pacific bluefin tuna, we implemented Bayes Factor Delimitation (BFD*; Leache et al. 2014), implemented in SNAPP (v1.3.0; Bryant et al. 2012), a package from BEAST (v2.4.7; Bouckaert et al. 2014). To do this, we generated a data set containing only Atlantic and Pacific bluefin tuna individuals, alongside bigeye tuna individuals as outgroups. We filtered this to include only one biallelic SNP (within the Pacific and Atlantic bluefin) with minor allele count >1 and with all individuals present per CORSET cluster. Delimitation runs were run for 48 steps at a chain length of 200,000 each, following 50,000 as pre-burn-in, with a gamma lambda prior of (*2, 200*). Two models were compared: 1) a model where individuals of Atlantic bluefin and Pacific bluefin corresponded to only one species, and 2) a model where individuals of Atlantic and Pacific bluefin correspond to two separate species (the current delimitation). Bigeye tuna was included as an outgroup in both analyses, designated as a separate species. Convergence was assessed by two separate runs of each model converging to within 1 log-likelihood unit. A Bayes Factor >10 was used to determine significance (Kass and Raftery 1995), and a posterior probability calculated by Bayes Factor/(Bayes Factor + 1).

### Timetree Inference

Using the concatenated transcript data set used in the supermatrix analysis and a fossil calibration, we estimated divergence dates for the *Thunnus* tuna. A hard-minimum fossil calibration of 37.8 My was used on the root of the tree based on the earliest known stem-group *Thunnus* fossil, *T. abchasicus*, which has been documented from the mid-Eocene in Russia (Monsch and Bannikov 2011). A soft-maximum age calibration of 56 My was used, corresponding to the start of the Eocene period. MCMCTree (v4.9e; Yang and Rannala 2006) was used for dating analysis. Following a burn-in of 100,000 iterations, Markov chains were sampled every 1,000th iteration until 40,000 trees were sampled, using the approximate likelihood algorithm. Priors for sigma2 and rgene were set to *G*(1, 10) and *G*(2, 11335) based on substitution rates inferred using BaseML. As the maximum-likelihood phylogenetic tree inferred from the same data set was relatively clock-like (root-to-tip variance 0.0000003) we used a global molecular clock to date the tree, following Walker et al. (2017). Two runs were carried out, with convergence of mean posterior times assessed, and infinite-site plots used to assess linearity of data (supplementary fig. S9, Supplementary Material online).

This dated tree was used to calculate EDGE scores (Isaac et al. 2007), based on IUCN red list threat-status (GE, as of February 2018; IUCN 2017) and evolutionary distinctness (ED) scores calculated in the R package “caper” (Orme 2013). EDGE scores for each species were calculated as follows: EDGE = ln(1+ED) + GE × ln(2).

### Genetic Structure

To assess genetic structure among the eight *Thunnus* species, we used an MDS and hierarchical clustering, using ADMIXTURE (v1.3; Alexander et al. 2009). Each *Thunnus* individual was regenotyped not allowing for homozygous blocks. Resultant vcf files were merged and filtered to include with at least one SNP, no indels, <5% missing taxa, and minor allele count >1 using vcftools (v0.1.3.2; Danecek et al. 2011). Using PLINK (v1.9b; Purcell et al. 2007), SNPs with an *R*^2^ value >0.1 of any other SNP within a 50-bp sliding window were removed to ensure unlinked SNPs were analyzed. ADMIXTURE runs were carried out with *k* values from 1 to 10, with the optimal run assessed using the lowest ADMIXTURE CV error. MDS analysis was carried out in PLINK.

### Tests for Introgression

To test whether a phylogenetic tree or a phylogenetic network, including hybridization events, best explains the data, we used a maximum pseudolikelihood approach (Solís-Lemus and Ané 2016), implemented within the Julia package PhyloNetworks (v0.6.0; Solís-Lemus et al. 2017). Uncollapsed transcript trees with >10 nodes with SH-like support >80 were used (only the transcript tree with the most such nodes per CORSET cluster was retained). Tip-based quartet concordance factors were calculated for each set of four individuals using the *readTrees2CF* function. Inter- and intraspecific concordance factors were then calculated from these using the *mapAllelesCFtable* function. Using SNaQ (Species Networks apply Quartets), a phylogenetic tree with no hybridization events was inferred. In order to assess whether the MSC adequately explains gene-tree discordance to this species tree, we used the TICR test (Tree Incongruence Checking in R; Stenz et al. 2015), using the “phylolm” R package. A chi-squared test was used to compare observed concordance factors with expected concordance factors calculated from the species tree under the MSC. Lack of significance (*P *>* *0.05) would indicate that the coalescent tree inferred without introgression events adequately fits the data.

To test whether the mitochondrial genome clustering of albacore and Pacific bluefin tuna is caused by introgression, we simulated gene trees under coalescence using “ms” (Hudson 2002), according to the coalescent units inferred by SNaQ. These coalescent units were unaltered, as they were first multiplied by 4, to account for the effective population size of the mitochondrial genome being one-fourth of the nuclear genome (Latta 2006), but then divided by 4, as coalescent units in SNaQ are defined as generations/effective population size, whereas in ms they are generations/4 × effective population size. In total, 100 replicates of 100,000 gene trees were simulated. The average frequency per replicate where Pacific bluefin and albacore clustered was used as a *P* value, with *P *<* *0.05 suggesting their clustering to be unlikely due to ILS alone (Buckley et al. 2006).

### Detecting Selection

We inferred selection on genetic variants in three groups: 1) the bluefin species; and 2) the warm-water species (yellowfin, bigeye, longtail, and blackfin); and 3) the visceral endotherm species (albacore, the three bluefin species, and bigeye). As they were monophyletic in the species tree, we tested for selection on de novo mutation in the warm-water tuna using the CodeML branch-site test (Zhang et al. 2005), within PAML v4.9e (Yang 2007). As they were not monophyletic, we tested for parallel selection on ancestral variation in the bluefin tunas and visceral endotherm tunas using a phylogenetic genome-wide association study (PhyloGWAS; Pease et al. 2016), implemented in MVFtools (v0.5.1.3; Pease and Rosenzweig 2015). Fixed sites for the longest CDS per CORSET cluster were used for all analyses.

For the CodeML branch-site test, genes whose gene trees significantly differed from the species tree (SH-test *P *<* *0.05) and in which the warm-water species were not monophyletic were discarded. Gene trees were used for those that significantly differed, but still had the warm-water species monophyletic. The species tree was used for the remainder of transcripts. For each CDS, four CodeML runs were carried out: a null model, where no site allows for ω > 1 in the target branch was compared with three runs of an alternate model, with an added site class allowing for ω > 1, each with different starting values of ω (0.5, 1, 1.5). Likelihood-ratio tests between each of these runs and the null were carried out, with significance inferred if *P *<* *0.05 in all three runs (χ^2^_1_). Gaps in the sequence data were allowed, but significant genes (*P *<* *0.05) where the associated nonsynonymous variants were present in only one of the warm-water species were discarded.

PhyloGWAS assesses whether there is an excess of nonsynonymous variants that are shared by individuals that are not monophyletic but share a phenotypic trait. Codon sites from all genes were filtered to remove sites with >2 nonsynonymous variants among the *Thunnus*, as these may reflect multiple changes rather than parallel selection on ancestral variation. Codon sites with >2 missing taxa were also filtered. The number of genes containing nonsynonymous mutations in the bluefin tunas and visceral endotherm tunas were calculated using MVFtools. Sites were only counted in the bluefin analysis if they had sequence data for each of the three bluefin species alongside the albacore tuna. Sites were only counted in the visceral endothermy analysis if they had sequence data for each of the three species lacking visceral endothermy (the longtail, blackfin, and yellowfin) alongside the bigeye tuna. To assess significance, this number was compared with the expected number shared due to ILS alone. To do this, 100,000,000 genes with a single change were simulated using ms (Hudson 2002) over the consensus phylogeny, using coalescent units inferred by SNaQ (these were divided by 4 as SNaQ coalescent units are generations/effective population size, whereas ms coalescent units are generations/4× effective population size). Two chromosomes (as tuna are diploid) were simulated for one individual of each species. The *P* values were the proportion of the simulated data sets that had at least the same number of shared substitutions as the observed number, out of a sample size the same as the number of variable amino acid sites tested. The number of variable amino acid sites was counted across all genes tested, including variable amino acids among species, which were previously filtered out when identifying fixed amino acids for each species. To allow for the codons with missing taxa in our data set, the number of sites in the simulated data set that fit the pattern except for one or two of the possible missing taxa was also counted, but weighted by the number of tested codons that had missing taxa.

GO term enrichment for genes under selection in each group was assessed using the topGO R package and the Fisher’s exact test with the “weight01” algorithm, and *P *<* *0.01 for significance (weight01 *P* values are deemed adjusted; Alexa et al. 2006). GO terms with only one representative in the significant set were discarded. Zebrafish orthologs for genes with functions relating to aerobic metabolism, which is hypothesized to relate to endothermy in bluefin tunas (Blank, Farwell, et al. 2007), were downloaded from UniProt (UniProt Consortium 2015). This was aligned with translated CDS from the tuna, using MAFFT (v7.271), and used to annotate which site the bluefin substitution was at. The same procedure was used for the warm-water tuna selection genes.

To examine possible functional effect of nonsynonymous variants, we predicted 3D protein structure for each of the identified candidate genes using the Phyre2 (Protein Homology/analogY Recognition Engine v2.0) webserver (http://www.sbg.bio.ic.ac.uk/phyre2; last accessed May 11, 2018; Kelley et al. 2015), using the “intensive” modeling mode. Prior to this, amino acids prior to the zebrafish start codon in the MAFFT alignment were trimmed. The evolutionary conservation of each nonsynonymous mutation was identified using the ConSurf webserver (http://consurf.tau.ac.il/2016; last accessed May 11, 2018). Slowly evolving regions are likely to have functional effects. Each amino acid site is therefore scored from 1 to 9, where 1 is highly variable and 9 is highly conserved (Ashkenazy et al. 2016). Effects of each mutation on protein stability were assessed using the SDM2 (Site Directed Mutator v2) webserver (http://structure.bioc.cam.ac.uk/sdm2; last accessed May 11, 2018; Pandurangan et al. 2017). Changes in electrostatic potential of each protein, which is responsible for catalytic activity in many enzymes, were measured using the MutantElec webserver (http://structuralbio.utalca.cl/mutantelec; last accessed May 11, 2018. Valdebenito-Maturana et al. 2017). MutantElec assesses whether amino acid changes significantly increase or decrease electrostatic potential using a nonparametric Wilcoxon signed-rank test with a confidence interval of 0.05.

## Supplementary Material

Supplementary data are available at *Molecular Biology and Evolution* online.

## Supplementary Material

Supplementary Data

## References

[msy198-B1] Aberer AJ, Kobert K, Stamatakis A. 2014 ExaBayes: massively parallel Bayesian tree inference for the whole-genome era. Mol Biol Evol. 31(10): 2553–2556. 25135941 10.1093/molbev/msu236 PMC4166930

[msy198-B2] Aken BL, Achuthan P, Akanni W, Amode MR, Bernsdorff F, Bhai J, Billis K, Carvalho-Silva D, Cummins C, Clapham P. 2017 Ensembl 2017. Nucleic Acids Res. 45(D1): D635–D642. 27899575 10.1093/nar/gkw1104 PMC5210575

[msy198-B3] Alexa A, Rahnenfuhrer J, Lengauer T. 2006 Improved scoring of functional groups from gene expression data by decorrelating GO graph structure. Bioinformatics 22(13): 1600–1607. 16606683 10.1093/bioinformatics/btl140

[msy198-B4] Alexander DH, Novembre J, Lange K. 2009 Fast model-based estimation of ancestry in unrelated individuals. Genome Res. 19(9): 1655–1664. 19648217 10.1101/gr.094052.109 PMC2752134

[msy198-B5] Altringham JD, Block BA. 1997 Why do tuna maintain elevated slow muscle temperatures? Power output of muscle isolated from endothermic and ectothermic fish. J Exp Biol. 200:2617–2627. 9359368 10.1242/jeb.200.20.2617

[msy198-B6] Altschul SF, Gish W, Miller W, Myers EW, Lipman DJ. 1990 Basic local alignment search tool. J Mol Biol. 215(3): 403–410. 2231712 10.1016/S0022-2836(05)80360-2

[msy198-B7] Anisimova M, Gil M, Dufayard J-F, Dessimoz C, Gascuel O. 2011 Survey of branch support methods demonstrates accuracy, power, and robustness of fast likelihood-based approximation schemes. Syst Biol. 60(5): 685–699. 21540409 10.1093/sysbio/syr041 PMC3158332

[msy198-B8] Arrizabalaga H, Dufour F, Kell L, Merino G, Ibaibarriaga L, Chust G, Irigoien X, Santiago J, Murua H, Fraile I. 2015 Global habitat preferences of commercially valuable tuna. Deep Sea Res Part II Top Stud Oceanogr. 113:102–112.

[msy198-B9] Ashkenazy H, Abadi S, Martz E, Chay O, Mayrose I, Pupko T, Ben-Tal N. 2016 ConSurf 2016: an improved methodology to estimate and visualize evolutionary conservation in macromolecules. Nucleic Acids Res. 44(W1): W344–W350. 27166375 10.1093/nar/gkw408 PMC4987940

[msy198-B10] Bateman A, Bennett HP. 1998 Granulins: the structure and function of an emerging family of growth factors. J Endocrinol. 158(2): 145–151. 9771457 10.1677/joe.0.1580145

[msy198-B11] Bayona-Vásquez NJ, Glenn TC, Uribe-Alcocer M, Pecoraro C, Díaz-Jaimes P. 2017 Complete mitochondrial genome of the yellowfin tuna (*Thunnus albacares*) and the blackfin tuna (*Thunnus atlanticus*): notes on mtDNA introgression and paraphyly on tunas. Conserv Genet Resour. 1–3.

[msy198-B12] Bernal D, Brill RW, Dickson KA, Shiels HA. 2017 Sharing the water column: physiological mechanisms underlying species-specific habitat use in tunas. Rev Fish Biol Fish. 27(4): 843–880.

[msy198-B13] Bernt M, Donath A, Jühling F, Externbrink F, Florentz C, Fritzsch G, Pütz J, Middendorf M, Stadler PF. 2013 MITOS: improved *de novo* metazoan mitochondrial genome annotation. Mol Phylogenet Evol. 69(2): 313–319. 22982435 10.1016/j.ympev.2012.08.023

[msy198-B14] Bestley S, Gunn JS, Hindell MA. 2009 Plasticity in vertical behaviour of migrating juvenile southern bluefin tuna (*Thunnus maccoyii*) in relation to oceanography of the south Indian Ocean. Fish Oceanogr. 18(4): 237–254.

[msy198-B15] Blank JM, Farwell CJ, Morrissette JM, Schallert RJ, Block BA. 2007 Influence of swimming speed on metabolic rates of juvenile pacific bluefin tuna and yellowfin tuna. Physiol Biochem Zool. 80(2): 167–177. 17252513 10.1086/510637

[msy198-B16] Blank JM, Morrissette JM, Farwell CJ, Price M, Schallert RJ, Block BA. 2007 Temperature effects on metabolic rate of juvenile Pacific bluefin tuna *Thunnus orientalis*. J Exp Biol. 210(Pt 23): 4254–4261. 18025023 10.1242/jeb.005835

[msy198-B17] Block BA, Finnerty JR. 1994 Endothermy in fishes: a phylogenetic analysis of constraints, predispositions, and selection pressures. Environ Biol Fishes. 40(3): 283–302.

[msy198-B18] Block BA, Finnerty JR, Stewart AFR, Kidd J. 1993 Evolution of endothermy in fish—mapping physiological traits on a molecular phylogeny. Science 260(5105): 210–214. 8469974 10.1126/science.8469974

[msy198-B19] Block BA, Jonsen ID, Jorgensen SJ, Winship AJ, Shaffer SA, Bograd SJ, Hazen EL, Foley DG, Breed GA, Harrison A-L, et al 2011 Tracking apex marine predator movements in a dynamic ocean. Nature 475(7354): 86–90. 21697831 10.1038/nature10082

[msy198-B20] Bonnet T, Leblois R, Rousset F, Crochet P-A. 2017 A reassessment of explanations for discordant introgressions of mitochondrial and nuclear genomes. Evolution 71(9): 2140–2158. 28703292 10.1111/evo.13296

[msy198-B21] Bouckaert R, Heled J, Kühnert D, Vaughan T, Wu C-H, Xie D, Suchard MA, Rambaut A, Drummond AJ. 2014 BEAST 2: a software platform for Bayesian evolutionary analysis. PLoS Comput Biol. 10(4): e1003537. 24722319 10.1371/journal.pcbi.1003537 PMC3985171

[msy198-B22] Breitburg D, Levin LA, Oschlies A, Grégoire M, Chavez FP, Conley DJ, Garçon V, Gilbert D, Gutiérrez D, Isensee K, et al 2018 Declining oxygen in the global ocean and coastal waters. Science 359:eaam7240. 29301986 10.1126/science.aam7240

[msy198-B23] Bryant D, Bouckaert R, Felsenstein J, Rosenberg NA, RoyChoudhury A. 2012 Inferring species trees directly from biallelic genetic markers: bypassing gene trees in a full coalescent analysis. Mol Biol Evol. 29(8): 1917–1932. 22422763 10.1093/molbev/mss086 PMC3408069

[msy198-B24] Buckley TR, Cordeiro M, Marshall DC, Simon C, Collins T. 2006 Differentiating between hypotheses of lineage sorting and introgression in New Zealand alpine cicadas (*Maoricicada* Dugdale). Syst Biol. 55:411–425. 16684720 10.1080/10635150600697283

[msy198-B25] Carey FG, Teal JM. 1966 Heat conservation in tuna fish muscle. Proc Natl Acad Sci U S A. 56(5): 1464–1469. 16591392 10.1073/pnas.56.5.1464 PMC220001

[msy198-B26] Cerveau N, Jackson DJ. 2016 Combining independent *de novo* assemblies optimizes the coding transcriptome for nonconventional model eukaryotic organisms. BMC Bioinformatics 17(1): 525. 27938328 10.1186/s12859-016-1406-x PMC5148890

[msy198-B27] Chang Z, Li G, Liu J, Zhang Y, Ashby C, Liu D, Cramer CL, Huang X. 2015 Bridger: a new framework for *de novo* transcriptome assembly using RNA-seq data. Genome Biol. 16:30. 25723335 10.1186/s13059-015-0596-2 PMC4342890

[msy198-B28] Chow S, Kishino H. 1995 Phylogenetic relationships between tuna species of the genus *Thunnus* (Scombridae: Teleostei): inconsistent implications from morphology, nuclear and mitochondrial genomes. J Mol Evol. 41(6): 741–748. 8587119 10.1007/BF00173154

[msy198-B29] Chow S, Nakagawa T, Suzuki N, Takeyama H, Matsunaga T. 2006 Phylogenetic relationships among *Thunnus* species inferred from rDNA ITS1 sequence. J Fish Biol. 68(A): 24–35.

[msy198-B30] Chung S, Zhou Z, Huddleston KA, Harrison DA, Reed R, Coleman TA, Rymond BC. 2002 Crooked neck is a component of the human spliceosome and implicated in the splicing process. Biochim Biophys Acta Gene Struct Expr. 1576(3): 287–297. 10.1016/s0167-4781(02)00368-8 12084575

[msy198-B31] Collette BB, Reeb C, Block BA. 2001 Systematics of the tunas and mackerels In: Block B, Stevens ED, editors. Tuna: physiology ecology and evolution. San Diego (CA): Academic Press p. 1–33.

[msy198-B32] Danecek P, Auton A, Abecasis G, Albers CA, Banks E, DePristo MA, Handsaker RE, Lunter G, Marth GT, Sherry ST, et al 2011 The variant call format and VCFtools. Bioinformatics 27(15): 2156–2158. 21653522 10.1093/bioinformatics/btr330 PMC3137218

[msy198-B33] Davidson NM, Oshlack A. 2014 Corset: enabling differential gene expression analysis for *de novo* assembled transcriptomes. Genome Biol. 15(7): 410. 25063469 10.1186/s13059-014-0410-6 PMC4165373

[msy198-B34] Díaz-Arce N, Arrizabalaga H, Murua H, Irigoien X, Rodríguez-Ezpeleta N. 2016 RAD-seq derived genome-wide nuclear markers resolve the phylogeny of tunas. Mol Phylogenet Evol. 102:202–207. 27286653 10.1016/j.ympev.2016.06.002

[msy198-B35] Dobin A, Davis CA, Schlesinger F, Drenkow J, Zaleski C, Jha S, Batut P, Chaisson M, Gingeras TR. 2013 STAR: ultrafast universal RNA-seq aligner. Bioinformatics 29(1): 15–21. 23104886 10.1093/bioinformatics/bts635 PMC3530905

[msy198-B36] Drost H-G, Paszkowski J. 2017 Biomartr: genomic data retrieval with R. Bioinformatics 33(8): 1216–1217. 28110292 10.1093/bioinformatics/btw821 PMC5408848

[msy198-B37] Durai DA, Schulz MH. 2016 Informed *k* mer selection for *de novo* transcriptome assembly. Bioinformatics 32(11): 1670–1677. 27153653 10.1093/bioinformatics/btw217 PMC4892416

[msy198-B38] FAO. 2016. The State of World Fisheries and Aquaculture 2016. SOFIA. Contributing to food security and nutrition for all, Rome: Food and Agriculture Organization, 2016, pp. 200.

[msy198-B39] Fitzgibbon QP, Baudinette RV, Musgrove RJ, Seymour RS. 2008 Routine metabolic rate of southern bluefin tuna *Thunnus maccoyii*. Comp Biochem Physiol A Mol Integr Physiol. 150(2): 231–238. 17081787 10.1016/j.cbpa.2006.08.046

[msy198-B40] Folk RA, Soltis PS, Soltis DE, Guralnick R. 2018 New prospects in the detection and comparative analysis of hybridization in the tree of life. Am J Bot. 105(3): 364–375. 29683488 10.1002/ajb2.1018

[msy198-B41] Fox T, DeBruin J, Haug Collet K, Trimnell M, Clapp J, Leonard A, Li B, Scolaro E, Collinson S, Glassman K, et al 2017 A single point mutation in *Ms44* results in dominant male sterility and improves nitrogen use efficiency in maize. Plant Biotechnol J. 15(8): 942–952. 28055137 10.1111/pbi.12689 PMC5506649

[msy198-B42] Fu L, Niu B, Zhu Z, Wu S, Li W. 2012 CD-HIT: accelerated for clustering the next-generation sequencing data. Bioinformatics 28(23): 3150–3152. 23060610 10.1093/bioinformatics/bts565 PMC3516142

[msy198-B43] Galli GLJ, Lipnick MS, Shiels HA, Block BA. 2011 Temperature effects on Ca^2+^ cycling in scombrid cardiomyocytes: a phylogenetic comparison. J Exp Biol. 214(7): 1068–1076. 21389190 10.1242/jeb.048231 PMC3052253

[msy198-B44] Gibbs RH, Collette BB. 1967 Comparative anatomy and systematics of the tunas, genus *Thunnus*. Fish Bull. 66:65–130.

[msy198-B45] Gong D-W, Bi S, Weintraub BD, Reitman M. 1998 Rat mitochondrial glycerol-3-phosphate dehydrogenase gene: multiple promoters, high levels in brown adipose tissue, and tissue-specific regulation by thyroid hormone. DNA Cell Biol. 17(3): 301–309. 9539110 10.1089/dna.1998.17.301

[msy198-B46] Good JM, Vanderpool D, Keeble S, Bi K. 2015 Negligible nuclear introgression despite complete mitochondrial capture between two species of chipmunks. Evolution 69(8): 1961–1972. 26118639 10.1111/evo.12712

[msy198-B47] Grabherr MG, Haas BJ, Yassour M, Levin JZ, Thompson DA, Amit I, Adiconis X, Fan L, Raychowdhury R, Zeng Q, et al 2011 Full-length transcriptome assembly from RNA-Seq data without a reference genome. Nat Biotechnol. 29(7): 644–652. 21572440 10.1038/nbt.1883 PMC3571712

[msy198-B48] Hahn MW, Nakhleh L. 2016 Irrational exuberance for resolved species trees. Evolution 70(1): 7–17. 26639662 10.1111/evo.12832

[msy198-B49] Hao Q, Yadav R, Basse AL, Petersen S, Sonne SB, Rasmussen S, Zhu Q, Lu Z, Wang J, Audouze K, et al 2015 Transcriptome profiling of brown adipose tissue during cold exposure reveals extensive regulation of glucose metabolism. Am J Physiol Metab. 308(5): E380–E392. 10.1152/ajpendo.00277.2014 25516548

[msy198-B50] Holland KN, Sibert JR. 1994 Physiological thermoregulation in bigeye tuna, *Thunnus obesus*. Environ Biol Fishes. 40(3): 319–327.

[msy198-B51] Hudson RR. 2002 Generating samples under a Wright-Fisher neutral model of genetic variation. Bioinformatics 18(2): 337–338. 11847089 10.1093/bioinformatics/18.2.337

[msy198-B52] Isaac NJB, Turvey ST, Collen B, Waterman C, Baillie JEM. 2007 Mammals on the EDGE: conservation priorities based on threat and phylogeny. PLoS One 2(3): e296. 17375184 10.1371/journal.pone.0000296 PMC1808424

[msy198-B53] ISC. 2016. Report of the Pacific Bluefin Tuna Working Group. La Jolla (CA): International Scientific Committee for Tuna and Tuna-Like Species in the North Pacific Ocean.

[msy198-B54] IUCN. 2017 The IUCN red list of threatened species. Version 2017-1. http://www.iucnredlist.org. Accessed on 2 December 2017.

[msy198-B55] Juan-Jordá MJ, Mosqueira I, Freire J, Dulvy NK. 2013 Life in 3-D: life history strategies in tunas, mackerels and bonitos. Rev Fish Biol Fish. 23(2): 135–155.

[msy198-B56] Juan-Jordá MJ, Mosqueira I, Freire J, Dulvy NK. 2015 Population declines of tuna and relatives depend on their speed of life. Proc R Soc Lond B Biol Sci. 282(1811): 20150322. 10.1098/rspb.2015.0322 PMC4528538 26156763

[msy198-B57] Kannan S, Hui J, Mazooji K, Pachter L, Tse D. 2016 Shannon: an information-optimal *de novo* RNA-Seq assembler. bioRxiv:039230.

[msy198-B58] Kass RE, Raftery AE. 1995 Bayes factors. J Am Stat Assoc. 90(430): 773–795.

[msy198-B59] Katoh K, Standley DM. 2013 MAFFT multiple sequence alignment software version 7: improvements in performance and usability. Mol Biol Evol. 30(4): 772–780. 23329690 10.1093/molbev/mst010 PMC3603318

[msy198-B60] Kelley LA, Mezulis S, Yates CM, Wass MN, Sternberg MJE. 2015 The Phyre2 web portal for protein modeling, prediction and analysis. Nat Protoc. 10(6): 845–858. 25950237 10.1038/nprot.2015.053 PMC5298202

[msy198-B61] Kroodsma DA, Mayorga J, Hochberg T, Miller NA, Boerder K, Ferretti F, Wilson A, Bergman B, White TD, Block BA, et al 2018 Tracking the global footprint of fisheries. Science 359(6378): 904–908. 29472481 10.1126/science.aao5646

[msy198-B62] Kubatko LS, Degnan JH, Collins T. 2007 Inconsistency of phylogenetic estimates from concatenated data under coalescence. Syst Biol. 56(1): 17–24. 17366134 10.1080/10635150601146041

[msy198-B63] Landeira-Fernandez AM, Morrissette JM, Blank JM, Block BA. 2004 Temperature dependence of the Ca^2+^-ATPase (SERCA2) in the ventricles of tuna and mackerel. Am J Physiol Regul Integr Comp Physiol. 286(2): R398–R404. 14604842 10.1152/ajpregu.00392.2003

[msy198-B64] Latta RG. 2006 Integrating patterns across multiple genetic markers to infer spatial processes. Landsc Ecol. 21(6): 809–820.

[msy198-B65] Leache AD, Fujita MK, Minin VN, Bouckaert RR. 2014 Species delimitation using genome-wide SNP data. Syst Biol. 63(4): 534–542. 24627183 10.1093/sysbio/syu018 PMC4072903

[msy198-B66] Li H. 2011 A statistical framework for SNP calling, mutation discovery, association mapping and population genetical parameter estimation from sequencing data. Bioinformatics 27(21): 2987–2993. 21903627 10.1093/bioinformatics/btr509 PMC3198575

[msy198-B67] Li H, Handsaker B, Wysoker A, Fennell T, Ruan J, Homer N, Marth G, Abecasis G, Durbin R. 2009 The Sequence Alignment/Map format and SAMtools. Bioinformatics 25(16): 2078–2079. 19505943 10.1093/bioinformatics/btp352 PMC2723002

[msy198-B68] Linthicum DS, Carey FG. 1972 Regulation of brain and eye temperatures by the bluefin tuna. Comp Biochem Physiol Part A Physiol. 43(2): 425–433. 10.1016/0300-9629(72)90201-0 4145250

[msy198-B69] Liu J, Li G, Chang Z, Yu T, Liu B, McMullen R, Chen P, Huang X. 2016 BinPacker: packing-based *de novo* transcriptome assembly from RNA-seq data. PLOS Comput Biol. 12(2): e1004772. 26894997 10.1371/journal.pcbi.1004772 PMC4760927

[msy198-B70] Lushchak OV, Piroddi M, Galli F, Lushchak VI. 2014 Aconitase post-translational modification as a key in linkage between Krebs cycle, iron homeostasis, redox signaling, and metabolism of reactive oxygen species. Redox Rep. 19(1): 8–15. 24266943 10.1179/1351000213Y.0000000073 PMC6837700

[msy198-B71] MacKenzie BR, Mosegaard H, Rosenberg AA. 2009 Impending collapse of bluefin tuna in the northeast Atlantic and Mediterranean. Conserv Lett. 2(1): 26–35.

[msy198-B72] Macmanes MD. 2014 On the optimal trimming of high-throughput mRNA sequence data. Front Genet. 5:13. 24567737 10.3389/fgene.2014.00013 PMC3908319

[msy198-B73] Maddison WP. 1997 Gene trees in species trees. Syst Biol. 46(3): 523–536.

[msy198-B74] Masson SWC, Hedges CP, Devaux JBL, James CS, Hickey AJR. 2017 Mitochondrial glycerol 3-phosphate facilitates bumblebee pre-flight thermogenesis. Sci Rep. 7(1): 13107. 29026172 10.1038/s41598-017-13454-5 PMC5638826

[msy198-B75] Matsuda C, Endo H, Ohta S, Kagawa Y. 1993 Gene structure of human mitochondrial ATP synthase gamma-subunit. Tissue specificity produced by alternative RNA splicing. J Biol Chem. 268(33): 24950–24958. 8227057

[msy198-B76] Matsuda H, Takenaka Y, Yahara T, Uozumi Y. 1998 Extinction risk assessment of declining wild populations: the case of the southern bluefin tuna. Res Popul Ecol (Kyoto). 40(3): 271–278.

[msy198-B77] Mattiazzi M, D'Aurelio M, Gajewski CD, Martushova K, Kiaei M, Beal MF, Manfredi G. 2002 Mutated human *SOD1* causes dysfunction of oxidative phosphorylation in mitochondria of transgenic mice. J Biol Chem. 277(33): 29626–29633. 12050154 10.1074/jbc.M203065200

[msy198-B78] McDonald AE, Pichaud N, Darveau C-A. 2018 “Alternative” fuels contributing to mitochondrial electron transport: importance of non-classical pathways in the diversity of animal metabolism. Comp Biochem Physiol B Biochem Mol Biol. 224:185–194. 10.1016/j.cbpb.2017.11.006 29155008

[msy198-B79] McKenna A, Hanna M, Banks E, Sivachenko A, Cibulskis K, Kernytsky A, Garimella K, Altshuler D, Gabriel S, Daly M, et al 2010 The Genome Analysis Toolkit: a MapReduce framework for analyzing next-generation DNA sequencing data. Genome Res. 20(9): 1297–1303. 20644199 10.1101/gr.107524.110 PMC2928508

[msy198-B80] Mendes FK, Hahn MW. 2017 Why concatenation fails near the anomaly zone. Syst Biol. 8:357–366. 10.1093/sysbio/syx063 28973673

[msy198-B81] Mirarab S, Reaz R, Bayzid MS, Zimmermann T, Swenson MS, Warnow T. 2014 ASTRAL: genome-scale coalescent-based species tree estimation. Bioinformatics 30(17): i541–i548. 25161245 10.1093/bioinformatics/btu462 PMC4147915

[msy198-B82] Miya M, Friedman M, Satoh TP, Takeshima H, Sado T, Iwasaki W, Yamanoue Y, Nakatani M, Mabuchi K, Inoue JG, et al 2013 Evolutionary origin of the scombridae (tunas and mackerels): members of a paleogene adaptive radiation with 14 other pelagic fish families. PLoS One 8(9): e73535. 24023883 10.1371/journal.pone.0073535 PMC3762723

[msy198-B83] Monsch KA, Bannikov AF. 2011 New taxonomic synopses and revision of the scombroid fishes (Scombroidei, Perciformes), including billfishes, from the Cenozoic of territories of the former USSR. Earth Environ Sci Trans R Soc Edinb. 102(04): 253–300.

[msy198-B84] Muhling BA, Lamkin JT, Alemany F, García A, Farley J, Ingram GW, Berastegui DA, Reglero P, Carrion RL. 2017 Reproduction and larval biology in tunas, and the importance of restricted area spawning grounds. Rev Fish Biol Fish. 27(4): 697–732.

[msy198-B85] Murphy MP. 2009 How mitochondria produce reactive oxygen species. Biochem J. 417(1): 1–13. 19061483 10.1042/BJ20081386 PMC2605959

[msy198-B86] Naiki M, Ochi N, Kato YS, Purevsuren J, Yamada K, Kimura R, Fukushi D, Hara S, Yamada Y, Kumagai T, et al 2014 Mutations in *HADHB*, which encodes the β-subunit of mitochondrial trifunctional protein, cause infantile onset hypoparathyroidism and peripheral polyneuropathy. Am J Med Genet A. 164(5): 1180–1187. 10.1002/ajmg.a.36434 24664533

[msy198-B87] Orme D. 2013 The caper package: comparative analysis of phylogenetics and evolution in R (v0.5.2). Available from: https://cran.r-project.org/web/packages/caper/index.html. Accessed on 2 December 2017.

[msy198-B88] Pamilo P, Nei M. 1988 Relationships between gene trees and species trees. Mol Biol Evol. 5(5): 568–583. 3193878 10.1093/oxfordjournals.molbev.a040517

[msy198-B89] Pandurangan AP, Ochoa-Montaño B, Ascher DB, Blundell TL. 2017 SDM: a server for predicting effects of mutations on protein stability. Nucleic Acids Res. 45(W1): W229–W235. 28525590 10.1093/nar/gkx439 PMC5793720

[msy198-B90] Pease J, Rosenzweig B. 2015 Encoding data using biological principles: the multisample variant format for phylogenomics and population genomics. IEEE/ACM Trans Comput Biol Bioinform. 10.1109/TCBB.2015.2509997 26701894

[msy198-B91] Pease JB, Haak DC, Hahn MW, Moyle LC. 2016 Phylogenomics reveals three sources of adaptive variation during a rapid radiation. PLoS Biol. 14(2): e1002379. 26871574 10.1371/journal.pbio.1002379 PMC4752443

[msy198-B92] Peng Y, Leung HCM, Yiu S-M, Lv M-J, Zhu X-G, Chin FYL. 2013 IDBA-tran: a more robust *de novo* de Bruijn graph assembler for transcriptomes with uneven expression levels. Bioinformatics 29(13): i326–i334. 23813001 10.1093/bioinformatics/btt219 PMC3694675

[msy198-B93] Pirie MD. 2015 Phylogenies from concatenated data: is the end nigh? Taxon 64(3): 421–423.

[msy198-B94] Pons J-M, Sonsthagen S, Dove C, Crochet P-A. 2014 Extensive mitochondrial introgression in North American Great Black-backed Gulls (*Larus marinus*) from the American Herring Gull (*Larus smithsonianus*) with little nuclear DNA impact. Heredity (Edinb) 112(3): 226–239. 24105440 10.1038/hdy.2013.98 PMC3931173

[msy198-B95] Purcell S, Neale B, Todd-Brown K, Thomas L, Ferreira MAR, Bender D, Maller J, Sklar P, de Bakker PIW, Daly MJ, et al 2007 PLINK: a tool set for whole-genome association and population-based linkage analyses. Am J Hum Genet. 81(3): 559–575. 17701901 10.1086/519795 PMC1950838

[msy198-B96] Qiu F, Kitchen A, Burleigh JG, Miyamoto MM. 2014 Scombroid fishes provide novel insights into the trait/rate associations of molecular evolution. J Mol Evol. 78(6): 338–348. 24810994 10.1007/s00239-014-9621-4

[msy198-B97] Robertson G, Schein J, Chiu R, Corbett R, Field M, Jackman SD, Mungall K, Lee S, Okada HM, Qian JQ, et al 2010 *De novo* assembly and analysis of RNA-seq data. Nat Methods. 7(11): 909–912. 20935650 10.1038/nmeth.1517

[msy198-B98] Safina C, Klinger DH. 2008 Collapse of bluefin tuna in the western Atlantic. Conserv Biol. 22(2): 243–246. 18402578 10.1111/j.1523-1739.2008.00901.x

[msy198-B99] Santini F, Carnevale G, Sorenson L. 2013 First molecular scombrid timetree (Percomorpha: Scombridae) shows recent radiation of tunas following invasion of pelagic habitat. Ital J Zool. 80(2): 210–221.

[msy198-B100] Sayyari E, Whitfield JB, Mirarab S. 2017 Fragmentary gene sequences negatively impact gene tree and species tree reconstruction. Mol Biol Evol. 34(12): 3279–3291. 29029241 10.1093/molbev/msx261

[msy198-B101] Schaefer KM. 2001 Reproductive biology of tunas In: Block BA, Stevens ED, editors. Tuna: physiology ecology and evolution. San Diego (CA): Academic Press p. 225–270.

[msy198-B102] Schaefer KM, Fuller DW. 2010 Vertical movements, behavior, and habitat of bigeye tuna (*Thunnus obesus*) in the equatorial eastern Pacific Ocean, ascertained from archival tag data. Mar Biol. 157(12): 2625–2642.

[msy198-B103] Schulz MH, Zerbino DR, Vingron M, Birney E. 2012 Oases: robust *de novo* RNA-seq assembly across the dynamic range of expression levels. Bioinformatics 28(8): 1086–1092. 22368243 10.1093/bioinformatics/bts094 PMC3324515

[msy198-B104] Sepulveda CA, Dickson KA, Bernal D, Graham JB. 2008 Elevated red myotomal muscle temperatures in the most basal tuna species, *Allothunnus fallai*. J Fish Biol. 73(1): 241–249.

[msy198-B105] Simão FA, Waterhouse RM, Ioannidis P, Kriventseva EV, Zdobnov EM. 2015 BUSCO: assessing genome assembly and annotation completeness with single-copy orthologs. Bioinformatics 31(19): 3210–3212. 26059717 10.1093/bioinformatics/btv351

[msy198-B106] Sloan DB, Havird JC, Sharbrough J. 2017 The on-again, off-again relationship between mitochondrial genomes and species boundaries. Mol Ecol. 26(8): 2212–2236. 27997046 10.1111/mec.13959 PMC6534505

[msy198-B107] Solís-Lemus C, Ané C. 2016 Inferring phylogenetic networks with maximum pseudolikelihood under incomplete lineage sorting. PLoS Genet. 12(3): e1005896. 26950302 10.1371/journal.pgen.1005896 PMC4780787

[msy198-B108] Solís-Lemus C, Bastide P, Ané C. 2017 PhyloNetworks: a package for phylogenetic networks. Mol Biol Evol. 34(12): 3292–3298. 28961984 10.1093/molbev/msx235

[msy198-B109] Song L, Florea L. 2015 Rcorrector: efficient and accurate error correction for Illumina RNA-seq reads. Gigascience 4:48. 26500767 10.1186/s13742-015-0089-y PMC4615873

[msy198-B110] Stamatakis A. 2014 RAxML version 8: a tool for phylogenetic analysis and post-analysis of large phylogenies. Bioinformatics 30(9): 1312–1313. 24451623 10.1093/bioinformatics/btu033 PMC3998144

[msy198-B111] Stenz NWM, Larget B, Baum DA, Ané C. 2015 Exploring tree-like and non-tree-like patterns using genome sequences: an example using the inbreeding plant species *Arabidopsis thaliana* (L.) Heynh. Syst Biol. 64(5): 809–823. 26117705 10.1093/sysbio/syv039

[msy198-B112] Stevens ED, Kanwisher JW, Carey F. 2000 Muscle temperature in free-swimming giant Atlantic bluefin tuna (*Thunnus thynnus* L.). J Therm Biol. 25(6): 419–423. 10880864 10.1016/s0306-4565(00)00004-8

[msy198-B113] Toews DPL, Brelsford A. 2012 The biogeography of mitochondrial and nuclear discordance in animals. Mol Ecol. 21(16): 3907–3930. 22738314 10.1111/j.1365-294X.2012.05664.x

[msy198-B114] UniProt Consortium. 2015 UniProt: a hub for protein information. Nucleic Acids Res. 43: D204–D212. 25348405 10.1093/nar/gku989 PMC4384041

[msy198-B115] Valdebenito-Maturana B, Reyes-Suarez JA, Henriquez J, Holmes DS, Quatrini R, Pohl E, Arenas-Salinas M. 2017 Mutantelec: an *in silico* mutation simulation platform for comparative electrostatic potential profiling of proteins. J Comput Chem. 38(7): 467–474. 28114729 10.1002/jcc.24712

[msy198-B116] Walker JF, Yang Y, Moore MJ, Mikenas J, Timoneda A, Brockington SF, Smith SA. 2017 Widespread paleopolyploidy, gene tree conflict, and recalcitrant relationships among the carnivorous Caryophyllales. Am J Bot. 104(6): 858–867. 28634254 10.3732/ajb.1700083

[msy198-B117] Wiens L, Banh S, Sotiri E, Jastroch M, Block BA, Brand MD, Treberg JR. 2017 Comparison of mitochondrial reactive oxygen species production of ectothermic and endothermic fish muscle. Front Physiol. 8:704. 28966595 10.3389/fphys.2017.00704 PMC5605635

[msy198-B118] Wilson SG, Jonsen ID, Schallert RJ, Ganong JE, Castleton MR, Spares AD, Boustany AM, Stokesbury MJW, Block BA. 2015 Tracking the fidelity of Atlantic bluefin tuna released in Canadian waters to the Gulf of Mexico spawning grounds. Can J Fish Aquat Sci. 72(11): 1700–1717.

[msy198-B300] Whitlock RE, Hazen EL, Walli A, Farwell C, Bograd SJ, Foley DG, Castleton M, Block BA. 2015 Direct quantification of energy intake in an apex marine predator suggests physiology is a key driver of migrations. Sci. Adv. 1(8):e1400270. 26601248 10.1126/sciadv.1400270 PMC4643779

[msy198-B119] Wu M, Kostyun JL, Hahn MW, Moyle L. 2017 Dissecting the basis of novel trait evolution in a radiation with widespread phylogenetic discordance. bioRxiv:201376:10.1111/mec.14780 29953708

[msy198-B120] Xie Y, Wu G, Tang J, Luo R, Patterson J, Liu S, Huang W, He G, Gu S, Li S, et al 2014 SOAPdenovo-Trans: *de novo* transcriptome assembly with short RNA-Seq reads. Bioinformatics 30(12): 1660–1666. 24532719 10.1093/bioinformatics/btu077

[msy198-B121] Yang Z. 2007 PAML 4: phylogenetic analysis by maximum likelihood. Mol Biol Evol. 24(8): 1586–1591. 17483113 10.1093/molbev/msm088

[msy198-B122] Yang Z, Rannala B. 2006 Bayesian estimation of species divergence times under a molecular clock using multiple fossil calibrations with soft bounds. Mol Biol Evol. 23(1): 212–226. 16177230 10.1093/molbev/msj024

[msy198-B123] Zerbino DR, Birney E. 2008 Velvet: algorithms for *de novo* short read assembly using de Bruijn graphs. Genome Res. 18(5): 821–829. 18349386 10.1101/gr.074492.107 PMC2336801

[msy198-B124] Zhang C, Rabiee M, Sayyari E, Mirarab S. 2018 ASTRAL-III: polynomial time species tree reconstruction from partially resolved gene trees. BMC Bioinformatics 19(Suppl 6): 153. 29745866 10.1186/s12859-018-2129-y PMC5998893

[msy198-B125] Zhang J, Nielsen R, Yang Z. 2005 Evaluation of an improved branch-site likelihood method for detecting positive selection at the molecular level. Mol Biol Evol. 22(12): 2472–2479. 16107592 10.1093/molbev/msi237

[msy198-B126] Zieliński P, Nadachowska-Brzyska K, Wielstra B, Szkotak R, Covaciu-Marcov SD, Cogălniceanu D, Babik W. 2013 No evidence for nuclear introgression despite complete mtDNA replacement in the Carpathian newt (*Lissotriton montandoni*). Mol Ecol. 22(7): 1884–1903. 23379646 10.1111/mec.12225

