# Supplementary material for: Phylotranscriptomic Insights into the Diversification of Endothermic Thunnus Tunas
Source: Mol Biol Evol. 2018 Oct 26;36(1):84–96. doi: 10.1093/molbev/msy198 (PMC6340463; doi:10.1093/molbev/msy198)
Supplement: Supplementary Data [file msy198_supp.docx]

# Supplementary table 1. Origin, read counts and mapping statistics for the 46 tuna individuals.

| Individual sample | Species | Origin | Tissue | Raw reads (paired) | Trimmed reads (paired), normalised if indicated by* | Read length (all paired-end) | Percentage of reads mapped against reference (unique, multi-mapped, total) | Number of SNPs called | Transcript present in with <50% gaps |
| --- | --- | --- | --- | --- | --- | --- | --- | --- | --- |
| PBFT1 | Pacific bluefin | Tuna Research and Conservation Centre, CA | Pooled red muscle, white muscle, atrium, spongy ventricle, compact ventricle | 169,487,057 | 24,687,074* | 100 | 36.71, 45.14, 81.85 | 47,164 | 33,833 |
| PBFT2 | Pacific bluefin | Tuna Research and Conservation Centre, CA | Pooled red muscle, white muscle, atrium, spongy ventricle, compact ventricle | 168,527,376 | 23,696,614* | 100 | 35.64, 45.39, 81.03 | 73,583 | 33,747 |
| PBFT3 | Pacific bluefin | Tuna Research and Conservation Centre, CA | Pooled white muscle, atrium, spongy ventricle, compact ventricle | 121,352,314 | 15,405,769* | 100 | 36.32, 46.03, 82.35 | 72,275 | 33,171 |
| PBFT4 | Pacific bluefin | Tuna Research and Conservation 100Centre, CA | Red muscle | 10,862,618 | 10,860,945 | 100 | 34.45, 44.65, 79.1 | 30,199 | 14,759 |
| PBFT5 | Pacific bluefin | Tuna Research and Conservation Centre, CA | Red muscle | 10,829,326 | 10,827,984 | 100 | 32.32, 47.07, 79.39 | 30,358 | 15,879 |
| PBFT6 | Pacific bluefin | Tuna Research and Conservation Centre, CA | Red muscle | 10,927,743 | 10,926,403 | 100 | 34.02, 44.57, 78.59 | 25,305 | 13,001 |
| PBFT7 | Pacific bluefin | Tuna Research and Conservation Centre, CA | White muscle | 10,907,030 | 10,905,417 | 100 | 23.89, 64.40, 88.29 | 22,277 | 13,387 |
| SBFT1 | Southern bluefin | [SRX10745](https://www.ncbi.nlm.nih.gov/sra/SRX1074515%5Baccn%5D)01 | Testis | 40,404,259 | 40,359,122 | 100 | 36.18, 37.51, 73.69 | 216,853 | 32,552 |
| SBFT2 | Southern bluefin | [SRX10745](https://www.ncbi.nlm.nih.gov/sra/SRX1074515%5Baccn%5D)02 | Testis | 39,407,512 | 39,362,418 | 100 | 36.91, 37.83, 74.74 | 209,635 | 32,462 |
| SBFT3 | Southern bluefin | [SRX10745](https://www.ncbi.nlm.nih.gov/sra/SRX1074515%5Baccn%5D)03 | Testis | 38,080,294 | 38,045,365 | 100 | 37.63, 40.3, 77.93 | 204,009 | 32,080 |
| SBFT4 | Southern bluefin | [SRX10745](https://www.ncbi.nlm.nih.gov/sra/SRX1074515%5Baccn%5D)04 | Testis | 41,415,688 | 41,374,855 | 100 | 38.2, 36.73, 74.93 | 199,600 | 32,011 |
| SBFT5 | Southern bluefin | [SRX10745](https://www.ncbi.nlm.nih.gov/sra/SRX1074515%5Baccn%5D)05 | Testis | 40,843,628 | 40,793,896 | 100 | 35.53, 36.86, 72.39 | 214,479 | 32,497 |
| SBFT6 | Southern bluefin | [SRX107451](https://www.ncbi.nlm.nih.gov/sra/SRX1074515%5Baccn%5D)1 | Ovary | 41,964,934 | 41,868,872 | 100 | 43.62, 42.36, 85.98 | 127,209 | 25,592 |
| SBFT7 | Southern bluefin | [SRX107451](https://www.ncbi.nlm.nih.gov/sra/SRX1074515%5Baccn%5D)2 | Ovary | 34,539,376 | 34,458,997 | 100 | 42.53, 42.56, 85.09 | 121,230 | 25,560 |
| SBFT8 | Southern bluefin | [SRX107451](https://www.ncbi.nlm.nih.gov/sra/SRX1074515%5Baccn%5D)3 | Ovary | 42,094,045 | 41,999,129 | 100 | 42.99, 43.48, 86.47 | 140,055 | 27,662 |
| SBFT9 | Southern bluefin | [SRX107451](https://www.ncbi.nlm.nih.gov/sra/SRX1074515%5Baccn%5D)4 | Ovary | 43,613,696 | 43,486,549 | 100 | 42.8, 42.59, 85.39 | 129,110 | 26,283 |
| SBFT10 | Southern bluefin | [SRX1074515](https://www.ncbi.nlm.nih.gov/sra/SRX1074515%5Baccn%5D) | Ovary | 40,380,684 | 40,309,497 | 100 | 43.8, 43.92, 87.72 | 135,891 | 27,144 |
| SBFT11 | Southern bluefin | SRX2255765 | White muscle | 53,932,658 | 53,931,508 | 100 | 25.48, 65.01, 90.49 | 104,234 | 22,843 |
| ABFT1 | Atlantic bluefin | SRX669379 | Liver | 21,329,510 | 21,328,787 | 101 | 37.92, 30.5, 68.42 | 39,921 | 20,025 |
| ABFT2 | Atlantic bluefin | SRX669391 | Liver | 21,005,851 | 21,004,919 | 101 | 40.39, 29.46, 69.85 | 41,966 | 20,791 |
| ABFT3 | Atlantic bluefin | SRX669406 | Liver | 25,210,795 | 25,209,670 | 101 | 38.01, 30.21, 68.22 | 44,599 | 21,456 |
| ABFT4 | Atlantic bluefin | SRX669993 | Kidney | 21,163,461 | 21,161,756 | 101 | 38.79, 41.84, 80.63 | 69,331 | 28,024 |
| ABFT5 | Atlantic bluefin | SRX669994 | Kidney | 27,064,384 | 27,062,367 | 101 | 37.73, 41.54, 79.27 | 73,935 | 28,709 |
| ABFT6 | Atlantic bluefin | SRX669995 | Kidney | 21,862,649 | 21,860,668 | 101 | 37.63, 43.11, 80.74 | 69,571 | 27,193 |
| ABFT7 | Atlantic bluefin | SRX2255758 | White muscle | 58,024,121 | 58,022,639 | 100 | 27.5, 65.71, 93.21 | 83,739 | 30,376 |
| BET1 | Bigeye | Purchased, super-frozen to -60C | White muscle | 19,242,071 | 19,241,080 | 100 | 21.11, 73.77, 94.88 | 64,596 | 16,054 |
| BET2 | Bigeye | Purchased, super-frozen to -60C | White muscle | 23,348,325 | 23,346,726 | 100 | 25.05, 69.03, 94.08 | 83,776 | 20,406 |
| BET3 | Bigeye | Purchased, super-frozen to -60C | White muscle | 21,967,433 | 21,965,874 | 100 | 21.42, 73.84, 95.26 | 63,115 | 15,420 |
| BET4 | Bigeye | SRX2255764 | White muscle | 59,947,884 | 59,946,833 | 100 | 24.23, 71.13, 95.36 | 144,306 | 28,952 |
| YFT1 | Yellowfin | Purchased | White muscle | 10,805,318 | 10,804,133 | 100 | 23.93, 64.51, 88.44 | 27,168 | 5,639 |
| YFT2 | Yellowfin | Purchased | White muscle | 10,865,984 | 10,864,755 | 100 | 28.27, 58.58, 86.85 | 31,501 | 6,447 |
| YFT3 | Yellowfin | Purchased | White muscle | 10,748,447 | 10,747,176 | 100 | 28.00, 58.41, 86.41 | 34,793 | 6,563 |
| YFT4 | Yellowfin | Tuna Research and Conservation Centre, CA | White muscle | 26,497,507 | 26,495,389 | 100 | 25.43, 70.13, 95.56 | 68,105 | 16,115 |
| YFT5 | Yellowfin | SRX2255763 | White muscle | 57,921,516 | 57,920,333 | 100 | 26.53, 68.57, 95.1 | 126,016 | 25,633 |
| Blackfin1 | Blackfin | Wild caught, Bahamas | White muscle | 26,467,969 | 26,467,159 | 100 | 28.59, 55.35, 83.94 | 34,409 | 2,934 |
| Blackfin2 | Blackfin | Wild caught, Bahamas | White muscle | 19,236,926 | 19,234,777 | 100 | 27.20, 46.11, 73.31 | 21,730 | 1,417 |
| Longtail1 | Longtail | Purchased | White muscle | 17,791,405 | 17,790,540 | 100 | 34.05, 44.10, 78.15 | 26,391 | 2,456 |
| Longtail2 | Longtail | Purchased | White muscle | 20,609,284 | 20,608,313 | 100 | 34.83, 19.28, 54.11 | 28,614 | 2,114 |
| Longtail3 | Longtail | Purchased | White muscle | 23,367,523 | 23,366,295 | 100 | 38.75, 21.06, 59.81 | 23,573 | 1,618 |
| Albacore1 | Albacore | Wild caught, Australia | White muscle | 10,838,535 | 10,837,106 | 100 | 24.14, 63.21, 87.85 | 47,759 | 9,133 |
| Albacore2 | Albacore | Purchased | White muscle | 10,901,682 | 10,900,166 | 100 | 26.99, 60.96, 87.95 | 44,607 | 7,539 |
| Albacore3 | Albacore | Purchased | White muscle | 10,849,034 | 10,847,482 | 100 | 28.79, 56.61, 85.4 | 56,038 | 10,357 |
| Albacore4 | Albacore | SRX2255762 | White muscle | 53,761,916 | 53,760,485 | 100 | 27.18, 65.75, 92.93 | 165,657 | 30,985 |
| Skipjack1 | Skipjack | Wild caught, Australia | White muscle | 11,716,680 | 11,714,851 | 100 | 25.16, 52.10, 77.26 | 265,245 | 10,326 |
| Skipjack2 | Skipjack | Wild caught, Australia | White muscle | 10,776,879 | 10,775,671 | 100 | 25.07, 54.24, 79.31 | 242,083 | 8,989 |
| Skipjack3 | Skipjack | SRX2255767 | White muscle | 59,325,533 | 59,324,751 | 100 | 29.09, 52.29, 81.38 | 631,110 | 21,882 |

Supplementary table 2. Assembly statistics for the 102 individual and 1 merged assemblies

| Assembly name (PBFT individual, assembly software, *k-*mer setting) | Number of contigs > 300 bp (Lowest indicated with **) | N50 (Highest indicated with **) | BUSCO completedness (%, highest indicated with **) | Number of contigs in final, clustered assembly (Highest indicated with **) |
| --- | --- | --- | --- | --- |
| PBFT1 Binpacker k19 | 93,557 | 1,999 | 27.1 | 829 |
| PBFT1 Binpacker k25 | 82,484 | 2,995 | 83.1 | 7,210** |
| PBFT1 Binpacker k32 | 74,904 | 3,287 | 85.9 | 2,485 |
| PBFT1 Bridger k19 | 96,300 | 2,133 | 26.7 | 305 |
| PBFT1 Bridger k25 | 89,815 | 2,955 | 83.1 | 520 |
| PBFT1 Bridger k32 | 83,203 | 3,256 | 85.8 | 237 |
| PBFT1 IDBA-trans k71 | 100,282 | 3,129 | 87.2 | 3,060 |
| PBFT1 OASES k21 | 149,619 | 2,970 | 79.6 | 1,519 |
| PBFT1 OASES k31 | 103,491 | 3,648 | 83.8 | 1,212 |
| PBFT1 OASES k41 | 85,736 | 3,788 | 83.6 | 845 |
| PBFT1 OASES k51 | 74,001 | 3,805 | 83.6 | 634 |
| PBFT1 OASES k61 | 67,047 | 3,747 | 81.2 | 527 |
| PBFT1 OASES k71 | 60,896 | 3,591 | 76.3 | 372 |
| PBFT1 Shannon k21 | 236,918 | 2,340 | 81.1 | 1,737 |
| PBFT1 Shannon k31 | 169,201 | 3,322 | 85.4 | 829 |
| PBFT1 Shannon k41 | 135,526 | 3,405 | 85.3 | 492 |
| PBFT1 Shannon k51 | 108,745 | 3,168 | 84.1 | 281 |
| PBFT1 Shannon k61 | 92,626 | 2,848 | 81.8 | 224 |
| PBFT1 Shannon k71 | 78,993 | 2,503 | 76.5 | 190 |
| PBFT1 SOAP-denovo-trans k21 | 68,366 | 1,990 | 77.7 | 324 |
| PBFT1 SOAP-denovo-trans k31 | 66,980 | 2,179 | 75.5 | 269 |
| PBFT1 SOAP-denovo-trans k41 | 70,006 | 1,841 | 69.2 | 250 |
| PBFT1 SOAP-denovo-trans k51 | 71,593 | 1,763 | 60.2 | 219 |
| PBFT1 SOAP-denovo-trans k61 | 70,875 | 1,748 | 55.1 | 187 |
| PBFT1 SOAP-denovo-trans k71 | 66,420 | 1,698 | 48.5 | 172 |
| PBFT1 transabyss k21 | 136,964 | 938 | 66.4 | 277 |
| PBFT1 transabyss k31 | 147,845 | 1,401 | 85.2 | 183 |
| PBFT1 transabyss k41 | 136,906 | 1,608 | 85.0 | 141 |
| PBFT1 transabyss k51 | 117,761 | 1,885 | 84.6 | 147 |
| PBFT1 transabyss k61 | 99,124 | 2,085 | 83.3 | 144 |
| PBFT1 transabyss k71 | 80,835 | 2,162 | 80.4 | 131 |
| PBFT1 trinity k19 | 147,289 | 1,072 | 55.4 | 313 |
| PBFT1 trinity k25 | 134,645 | 2,466 | 78.4 | 418 |
| PBFT1 trinity k32 | 126,086 | 2,804 | 80.1 | 293 |
| PBFT2 Binpacker k19 | 91,237 | 2,006 | 25.7 | 411 |
| PBFT2 Binpacker k25 | 79,564 | 2,977 | 81.1 | 2,363 |
| PBFT2 Binpacker k32 | 72,749 | 3,312 | 83.5 | 967 |
| PBFT2 Bridger k19 | 94,155 | 2,126 | 26.7 | 242 |
| PBFT2 Bridger k25 | 87,464 | 2,922 | 80.9 | 285 |
| PBFT2 Bridger k32 | 81,279 | 3,291 | 83.4 | 100 |
| PBFT2 IDBA-trans k71 | 97,964 | 3,152 | 84.4 | 1,161 |
| PBFT2 OASES k21 | 142,087 | 3,099 | 77.6 | 742 |
| PBFT2 OASES k31 | 97,847 | 3,802 | 80.9 | 568 |
| PBFT2 OASES k41 | 82,212 | 3,916 | 80.7 | 459 |
| PBFT2 OASES k51 | 71,711 | 3,876 | 79.2 | 340 |
| PBFT2 OASES k61 | 64,916 | 3,836 | 76.1 | 340 |
| PBFT2 OASES k71 | 58,475 | 3,740 | 71.0 | 289 |
| PBFT2 Shannon k21 | 235,196 | 2,372 | 78.5 | 1,218 |
| PBFT2 Shannon k31 | 168,069 | 3,386 | 82.7 | 588 |
| PBFT2 Shannon k41 | 133,849 | 3,430 | 81.6 | 377 |
| PBFT2 Shannon k51 | 107,587 | 3,193 | 80.0 | 213 |
| PBFT2 Shannon k61 | 92,452 | 2,913 | 76.9 | 173 |
| PBFT2 Shannon k71 | 78,458 | 2,558 | 71.2 | 142 |
| PBFT2 SOAP-denovo-trans k21 | 66,950 | 1,963 | 74.7 | 260 |
| PBFT2 SOAP-denovo-trans k31 | 65,322 | 2,144 | 73.2 | 191 |
| PBFT2 SOAP-denovo-trans k41 | 66,974 | 1,955 | 68.2 | 174 |
| PBFT2 SOAP-denovo-trans k51 | 71,968 | 1,544 | 57.9 | 164 |
| PBFT2 SOAP-denovo-trans k61 | 68,822 | 1,705 | 52.2 | 129 |
| PBFT2 SOAP-denovo-trans k71 | 64,432 | 1,665 | 45.6 | 112 |
| PBFT2 transabyss k21 | 133,725 | 943 | 65.5 | 223 |
| PBFT2 transabyss k31 | 143,999 | 1,422 | 82.5 | 151 |
| PBFT2 transabyss k41 | 133,143 | 1,620 | 82.2 | 106 |
| PBFT2 transabyss k51 | 115,201 | 1,877 | 81.1 | 127 |
| PBFT2 transabyss k61 | 97,749 | 2,058 | 79.6 | 111 |
| PBFT2 transabyss k71 | 79,619 | 2,151 | 75.7 | 102 |
| PBFT2 trinity k19 | 144,294 | 1,077 | 54.0 | 301 |
| PBFT2 trinity k25 | 129,797 | 2,568 | 77.3 | 353 |
| PBFT2 trinity k32 | 120,768 | 2,908 | 78.8 | 226 |
| PBFT3 Binpacker k19 | 76,305 | 1,859 | 28.8 | 386 |
| PBFT3 Binpacker k25 | 64,656 | 2,690 | 74.2 | 1,493 |
| PBFT3 Binpacker k32 | 59,509 | 2,895 | 75.4 | 465 |
| PBFT3 Bridger k19 | 79,125 | 1,923 | 28.5 | 216 |
| PBFT3 Bridger k25 | 70,743 | 2,641 | 73.9 | 133 |
| PBFT3 Bridger k32 | 65,894 | 2,849 | 75.4 | 34 |
| PBFT3 IDBA-trans k71 | 78,255 | 2,772 | 77.4 | 539 |
| PBFT3 OASES k21 | 115,029 | 2,817 | 72.2 | 441 |
| PBFT3 OASES k31 | 81,077 | 3,059 | 72.5 | 294 |
| PBFT3 OASES k41 | 65,433 | 3,276 | 72.2 | 275 |
| PBFT3 OASES k51 | 56,931 | 3,195 | 69.4 | 230 |
| PBFT3 OASES k61 | 51,912 | 3,069 | 63.8 | 164 |
| PBFT3 OASES k71 | 45,614 | 2,881 | 54.8 | 169 |
| PBFT3 Shannon k21 | 163,485 | 2,212 | 72.5 | 691 |
| PBFT3 Shannon k31 | 114,066 | 2,931 | 74.9 | 249 |
| PBFT3 Shannon k41 | 95,814 | 2,859 | 73.0 | 181 |
| PBFT3 Shannon k51 | 80,470 | 2,551 | 69.4 | 99 |
| PBFT3 Shannon k61 | 72,366 | 2,245 | 63.5 | 108 |
| PBFT3 Shannon k71 | 62,036 | 1,890 | 54.3 | 89 |
| PBFT3 SOAP-denovo-trans k21 | 55,456 | 1,828 | 67.3 | 147 |
| PBFT3 SOAP-denovo-trans k31 | 54,560 | 1,862 | 63.8 | 154 |
| PBFT3 SOAP-denovo-trans k41 | 55,820 | 1,652 | 58.9 | 121 |
| PBFT3 SOAP-denovo-trans k51 | 56,594 | 1,556 | 52.2 | 109 |
| PBFT3 SOAP-denovo-trans k61 | 55,834 | 1,463 | 43.8 | 89 |
| PBFT3 SOAP-denovo-trans k71 | 50,696 | 1,365 | 36.4 | 71 |
| PBFT3 transabyss k21 | 103,693 | 1,001 | 61.6 | 142 |
| PBFT3 transabyss k31 | 105,704 | 1,445 | 74.9 | 88 |
| PBFT3 transabyss k41 | 97,809 | 1,605 | 73.7 | 67 |
| PBFT3 transabyss k51 | 83,794 | 1,803 | 71.5 | 49 |
| PBFT3 transabyss k61 | 71,707 | 1,900 | 67.8 | 70 |
| PBFT3 transabyss k71 | 59,375 | 1,871 | 61.4 | 71 |
| PBFT3 trinity k19 | 107,323 | 1,234 | 57.1 | 236 |
| PBFT3 trinity k25 | 103,030 | 2,168 | 67.0 | 165 |
| PBFT3 trinity k32 | 96,649 | 2,408 | 68.8 | 129 |
| Merge Assembly | 48,648** | 4,268** | 89.1** | - |

Supplementary table 3. Genes with fixed non-synonymous mutations shared by the three bluefin tuna species. ENSEMBL gene id followed by the top blastp result against the nr database is supplied when an annotation was not available for the ENSEMBL gene.

| Transcript ID | Top blast hit gene | Gene symbol | Number of non-synonymous mutations | Number of synonymous mutations | Gene Ontology terms | Gene ontology terms enriched, or with hypothesised bluefin function? |
| --- | --- | --- | --- | --- | --- | --- |
| PBFT1_Binpacker_25_BINPACKER.14495.1 | Glycosylated lysosomal membrane protein | *GLMP* | 2 | 1 | integral component of membrane, membrane |  |
| PBFT1_Binpacker_25_BINPACKER.7210.1 | Coiled-coil domain containing *137* | *CCDC137* | 1 | 0 | - |  |
| PBFT2_OASES_61_Locus_54116_Transcript_1_1_Confidence_1.000_Length_2558 | Diacylglycerol O-acyltransferase *2* | *DGAT2* | 2 | 0 | integral component of membrane, membrane, transferase activity, transferring acyl groups other than amino-acyl groups |  |
| PBFT1_transabyss_21_R3087861 | Interferon, gamma-inducible protein *30* | *IFI30* | 2 | 0 | oxidation-reduction process |  |
| PBFT1_Binpacker_25_BINPACKER.5154.1 | Eukaryotic translation initiation factor *3*, subunit *10* (theta) | *EIF3S10* | 2 | 0 | eukaryotic 48S preinitiation complex, eukaryotic 43S preinitiation complex, eukaryotic translation initiation factor 3 complex, regulation of translational initiation, formation of translation preinitiation complex, translational initiation, translation, translation initiation factor activity, RNA binding, cytoplasm |  |
| PBFT1_Binpacker_32_BINPACKER.8851.1 | Charged multivesicular body protein *5*b | *CHMP5B* | 2 | 0 | vacuolar transport, intracellular |  |
| PBFT1_OASES_21_Locus_22080_Transcript_2_6_Confidence_0.722_Length_4332 | ENSTRUP00000013522: Spermatogenesis-associated protein 2-like | *SPATA2L* | 1 | 0 | - |  |
| PBFT1_Binpacker_32_BINPACKER.2708.1 | Adaptor related protein complex *3* beta *1* subunit | *AP3B1* | 2 | 0 | clathrin adaptor complex, AP-3 adaptor complex, membrane coat, protein transport, transport, vesicle-mediated transport, intracellular protein transport, membrane, clathrin adaptor complex, AP-3 adaptor complex, membrane coat, protein transport, transport, vesicle-mediated transport, intracellular protein transport, membrane |  |
| PBFT2_Binpacker_25_BINPACKER.43.1 | Superoxide dismutase *1*, soluble | *SOD1* | 2 | 0 | neuron cellular homeostasis, response to methylmercury, response to metal ion, response to xenobiotic stimulus, superoxide dismutase activity, removal of superoxide radicals, superoxide metabolic process, zinc ion binding, oxidation-reduction process, oxidoreductase activity, cytoplasm, copper ion binding, extracellular space, metal ion binding | Yes, superoxidase activity |
| PBFT3_OASES_21_Locus_6265_Transcript_10_10_Confidence_0.118_Length_3198 | Aryl hydrocarbon receptor *2*C, mRNA | *AHR2C* | 1 | 2 | nuclear aryl hydrocarbon receptor complex, aryl hydrocarbon receptor complex, positive regulation of transcription from RNA polymerase II promoter, intracellular receptor signaling pathway, response to xenobiotic stimulus, aryl hydrocarbon receptor activity, protein dimerization activity, nucleus, regulation of transcription, DNA-templated, transcription, DNA-templated, transcription factor activity, sequence-specific DNA binding |  |
| PBFT2_Binpacker_25_BINPACKER.6252.2 | ENSONIP00000024784.1: PREDICTED: ATPase inhibitor, mitochondrial | *ATPIF1* | 1 | 2 | negative regulation of ATPase activity, ATPase inhibitor activity, mitochondrion |  |
| PBFT1_SOAP_61_scaffold19122 | ENSONIP00000016193.1: PREDICTED: APC membrane recruitment protein 1 | *AMER1* | 1 | 1 | negative regulation of Wnt signaling pathway |  |
| PBFT1_Binpacker_25_BINPACKER.7206.1 | *SLU7* homolog, splicing factor | *SLU7* | 1 | 1 | zinc ion binding, nucleic acid binding |  |
| PBFT1_Binpacker_25_BINPACKER.20261.1 | Solute carrier family *17* member *3* | *SLC17A3* | 1 | 1 | transmembrane transport, integral component of membrane, membrane |  |
| PBFT2_OASES_31_Locus_5894_Transcript_4_8_Confidence_0.550_Length_7090 | proteasome activator subunit *4a* | *PSME4A* | 1 | 0 | peptidase activator activity, positive regulation of peptidase activity, proteasome binding, lysine-acetylated histone binding |  |
| PBFT1_OASES_21_Locus_8818_Transcript_6_8_Confidence_0.611_Length_3192 | *SUV3*-like helicase | *SUPV3L1* | 1 | 0 | hydrolase activity, acting on acid anhydrides |  |
| PBFT1_Binpacker_25_BINPACKER.6874.1 | *SPT2* chromatin protein domain containing *1* | *SPTY2D1* | 1 | 0 | - |  |
| PBFT1_Binpacker_25_BINPACKER.2784.1 | A-Raf proto-oncogene, serine/threonine kinase | *ARAF* | 1 | 0 | ATP binding, nucleotide binding, signal transduction, phosphorylation, protein phosphorylation, kinase activity, protein kinase activity, intracellular signal transduction, metal ion binding, intracellular, transferase activity, signal transducer activity, downstream of receptor, protein serine/threonine kinase activity, SMAD binding, pathway-restricted SMAD protein phosphorylation, negative regulation of nodal signaling pathway |  |
| PBFT1_Binpacker_25_BINPACKER.8444.2 | Solute carrier family *14* (urea transporter), member *2* | *SLC14A2* | 1 | 0 | urea transport, urea transmembrane transport, urea transmembrane transporter activity, integral component of membrane, membrane |  |
| PBFT1_SOAP_41_C574394 | Glycerol-*3*-phosphate dehydrogenase *1b* | *GPD1B* | 1 | 0 | glycerol-3-phosphate dehydrogenase complex, glycerol-3-phosphate catabolic process, glycerol-3-phosphate metabolic process, glycerol-3-phosphate dehydrogenase [NAD+] activity, protein homodimerization activity, carbohydrate metabolic process, oxidoreductase activity, NAD binding, oxidation-reduction process, oxidoreductase activity, acting on the CH-OH group of donors, NAD or NADP as acceptor | Yes, GO terms enriched |
| PBFT3_Bridger_32_comp3079_seq2 | Lysine (K)-specific demethylase *5Ba* | *KDM5BA* | 1 | 0 | metal ion binding, DNA binding, nucleus |  |
| PBFT2_OASES_21_Locus_501_Transcript_3_3_Confidence_0.906_Length_3140 | WD repeat domain *3* | *WDR3* | 1 | 0 | - |  |
| PBFT2_Binpacker_32_BINPACKER.13269.4 | Integrin, alpha *11b* | *ITGA11B* | 1 | 0 | integrin complex, integrin-mediated signaling pathway, cell adhesion, membrane, integral component of membrane |  |
| PBFT1_Binpacker_25_BINPACKER.17228.1 | Trypsin domain containing *1* | *TYSND1* | 1 | 0 | serine-type peptidase activity, proteolysis, hydrolase activity, peptidase activity |  |
| PBFT2_Shannon_21_Shannon_PBFT2_k21_c1_77_10360_1036 | *NSFL1* (p*97*) cofactor (p*47*) | *NSFL1C* | 1 | 0 | - |  |
| PBFT2_IDBA_71_transcript-71_47409 | RNA *3*'-terminal phosphate cyclase | *RTCA* | 1 | 0 | RNA-3'-phosphate cyclase activity, RNA processing, catalytic activity, ATP binding, nucleotide binding, RNA-3'-phosphate cyclase activity, RNA processing, catalytic activity, ATP binding, nucleotide binding |  |
| PBFT2_transabyss_51_J764135 | Ribosomal protein *L6* | *RPL6* | 1 | 0 | chordate embryonic development, pancreas development, intracellular, ribosome, translation, structural constituent of ribosome |  |
| PBFT2_OASES_51_Locus_5596_Transcript_1_2_Confidence_0.750_Length_5546 | Adenosine deaminase, RNA-specific | *ADAR* | 1 | 0 | RNA binding, RNA processing, adenosine deaminase activity, double-stranded RNA adenosine deaminase activity |  |
| PBFT1_Binpacker_19_BINPACKER.53.5 | Syntaxin binding protein *3* | *STXBP3* | 1 | 0 | vesicle docking involved in exocytosis, vesicle-mediated transport |  |
| PBFT3_Binpacker_32_BINPACKER.590.6 | ENSPFOP00000022026.1: PREDICTED: neurofilament heavy polypeptide-like isoform X4 | *NEFH* | 1 | 0 | sarcoplasmic reticulum, receptor binding, integral component of membrane, membrane |  |
| PBFT3_IDBA_71_transcript-71_19698 | WW domain binding protein 1-like b | *WBP1LB* | 1 | 0 | integral component of membrane, membrane |  |
| PBFT2_OASES_71_Locus_11217_Transcript_1_6_Confidence_0.231_Length_1616 | ENSPFOP00000022539.1: PREDICTED: CAP-Gly domain-containing linker protein 1-like isoform X3 | *CLIP1* | 1 | 0 | - |  |
| PBFT1_Binpacker_32_BINPACKER.6704.2 | Large *60*S subunit nuclear export GTPase *1* | *LSG1* | 1 | 0 | GTP binding |  |
| PBFT1_transabyss_61_R548547 | Phosphorylated adaptor for RNA export | *PHAX* | 1 | 0 | - |  |
| PBFT2_transabyss_51_R755216 | ENSPFOP00000028504.1: PREDICTED: CREB3 regulatory factor-like | *CREBRF* | 1 | 0 | regulation of transcription, DNA-templated, transcription factor activity, sequence-specific DNA binding |  |
| PBFT1_Trinity_25_TRINITY_DN27436_c0_g1_i2 | Aldehyde dehydrogenase *9* family, member A*1*b | *ALDH9A1B* | 1 | 0 | oxidoreductase activity, oxidation-reduction process, metabolic process |  |
| PBFT1_Binpacker_25_BINPACKER.8827.1 | WD repeat domain *1* | *WDR1* | 1 | 0 | - |  |
| PBFT1_OASES_71_Locus_9404_Transcript_1_2_Confidence_0.667_Length_1769 | Exosome component *9* | *EXOSC9* | 1 | 0 | exosome (RNase complex), RNA processing |  |
| PBFT1_OASES_61_Locus_16320_Transcript_1_3_Confidence_0.667_Length_1472 | Replication factor C (activator *1*) *5* | *RFC5* | 1 | 0 | retina development in camera-type eye, DNA replication, DNA binding, ATP binding |  |
| PBFT1_Binpacker_25_BINPACKER.6801.5 | Fibrosin | *FBRS* | 1 | 0 | - |  |
| PBFT1_Shannon_31_Shannon_PBFT1_k31_cremaining39_30788_0 | *CWF19*-like *2*, cell cycle control | *CWF19L2* | 1 | 0 | - |  |
| PBFT1_Binpacker_25_BINPACKER.15103.1 | Ubiquitin specific peptidase *45* | *USP45* | 1 | 0 | thiol-dependent ubiquitinyl hydrolase activity, zinc ion binding, proteolysis, hydrolase activity, peptidase activity, cysteine-type peptidase activity, metal ion binding, protein deubiquitination, ubiquitin-dependent protein catabolic process |  |
| PBFT2_IDBA_71_transcript-71_8846 | ENSXMAP00000006515.1: low-density lipoprotein receptor-like | *LDLR* | 1 | 0 | calcium ion binding, integral component of membrane, membrane |  |
| PBFT2_OASES_31_Locus_2354_Transcript_4_7_Confidence_0.567_Length_4823 | Apoptosis-inducing factor, mitochondrion-associated *1* | *AIFM1* | 1 | 0 | flavin adenine dinucleotide binding, oxidoreductase activity, protein dimerization activity, oxidation-reduction process |  |
| PBFT2_SOAP_21_scaffold22529 | U6 snRNA biogenesis *1* | *USB1* | 1 | 0 | regulation of RNA splicing, U6 snRNA 3'-end processing, neutrophil differentiation, RNA splicing, mRNA processing, nuclease activity, hydrolase activity, nucleic acid phosphodiester bond hydrolysis, nucleus, hemopoiesis |  |
| PBFT2_Shannon_41_Shannon_PBFT2_k41_c1_74_1983_1 | Aconitase *2* | *ACO2* | 1 | 0 | aconitate hydratase activity, 4 iron, 4 sulfur cluster binding, iron-sulfur cluster binding, lyase activity, metabolic process, mitochondrion, tricarboxylic acid cycle, metal ion binding | Yes, aerobic metabolism |
| PBFT1_OASES_61_Locus_19726_Transcript_1_2_Confidence_0.750_Length_2154 | RNA polymerase II associated protein *2* | *RPAP2* | 1 | 0 | - |  |
| PBFT3_OASES_21_Locus_6664_Transcript_3_3_Confidence_0.778_Length_1227 | Stomatin (EPB72)-like *3*b | *STOML3B* | 1 | 0 | membrane, integral component of membrane |  |
| PBFT1_IDBA_71_transcript-71_16283 | ENSPFOP00000028627.1: PREDICTED: fibrous sheath CABYR-binding protein-like isoform X5 | *FSCB* | 1 | 0 | sarcoplasmic reticulum, receptor binding, integral component of membrane, membrane |  |
| PBFT1_OASES_51_Locus_9379_Transcript_6_7_Confidence_0.450_Length_4795 | tubulin alpha *4*a | *TUBA4A* | 1 | 0 | microtubule-based process, GTPase activity, microtubule, structural constituent of cytoskeleton, cytoskeleton organization, cytoskeleton, GTP binding, cytoplasm, nucleotide binding |  |
| PBFT1_Binpacker_25_BINPACKER.18421.1 | Phosphoinositide *5*-phosphatase | *FIG4* | 1 | 0 | phosphoric ester hydrolase activity |  |
| PBFT1_Binpacker_19_BINPACKER.49.1 | Leucine-rich, glioma inactivated *1b* | *LGI1B* | 1 | 0 | myelination, brain morphogenesis |  |
| PBFT1_IDBA_71_transcript-71_22514 | *TBC1* domain family, member *10b* | *TBC1D10B* | 1 | 0 | - |  |
| PBFT1_Binpacker_25_BINPACKER.16707.1 | *NACC* family member *2* | *NACC2* | 1 | 0 | - |  |
| PBFT2_OASES_71_Locus_29943_Transcript_1_1_Confidence_1.000_Length_1739 | Flavin adenine dinucleotide synthetase *1* | *FLAD1* | 1 | 0 | FMN adenylyltransferase activity, FAD biosynthetic process, nucleotidyltransferase activity, catalytic activity, ATP binding, nucleotide binding, metabolic process, transferase activity |  |
| PBFT1_Binpacker_25_BINPACKER.8465.1 | Solute carrier family *1* (neutral amino acid transporter), member *5* | *SLC1A5* | 1 | 0 | transport, integral component of membrane, membrane, symporter activity, transmembrane transport |  |
| PBFT3_OASES_61_Locus_2835_Transcript_1_2_Confidence_0.667_Length_1122 | *NSA2* ribosome biogenesis homolog | *NSA2* | 1 | 0 | - |  |
| PBFT3_Shannon_21_Shannon_PBFT3_k21_r2_c1_99_5160_516 | ENSONIP00000022928.1: PREDICTED: calcium-binding and coiled-coil domain-containing protein 2 | *CALCOCO2* | 1 | 0 | - |  |
| PBFT1_Binpacker_25_BINPACKER.5077.1 | Ring finger protein *141* | *RNF141* | 1 | 0 | - |  |
| PBFT1_Binpacker_25_BINPACKER.18996.1 | Non-SMC condensin II complex, subunit *G2* | *NCAPG2* | 1 | 0 | nucleus |  |
| PBFT1_Binpacker_25_BINPACKER.35078.1 | Collagen type XIX alpha *1* chain | *COL19A1* | 1 | 0 | - |  |
| PBFT2_SOAP_61_scaffold21807 | *SEC22* homolog B, vesicle trafficking protein (gene/pseudogene) | *SEC22B* | 1 | 0 | vesicle fusion with Golgi apparatus, ER to Golgi vesicle-mediated transport, SNARE complex, vesicle-mediated transport, SNAP receptor activity, SNARE binding, integral component of membrane, membrane |  |
| PBFT1_Binpacker_32_BINPACKER.10017.2 | Integrator complex subunit *1* | *INTS1* | 1 | 0 | - |  |
| PBFT3_OASES_21_Locus_3418_Transcript_6_10_Confidence_0.661_Length_6199 | ENSPFOP00000030596.1: PREDICTED: titin homolog isoform X1 | *TTN1* | 1 | 0 | - |  |
| PBFT3_Binpacker_25_BINPACKER.5812.1 | Testis-specific kinase *2* | *TESK2* | 1 | 0 | protein phosphorylation, protein kinase activity, ATP binding |  |
| PBFT2_Binpacker_25_BINPACKER.7517.2 | Trinucleotide repeat containing *6C1* | *TNRC6C1* | 1 | 0 | miRNA mediated inhibition of translation, nucleic acid binding |  |
| PBFT1_Shannon_21_Shannon_PBFT1_k21_c1_42_137190_13719 | *S100* calcium binding protein U | *S100U* | 1 | 0 | copper ion binding, transition metal ion binding, metal ion binding, calcium ion binding |  |
| PBFT3_OASES_71_Locus_4663_Transcript_1_1_Confidence_1.000_Length_2544 | Nuclear factor, interleukin I regulated, member *5* | *NFIL3-5* | 1 | 0 | transcription factor activity, sequence-specific DNA binding, regulation of transcription, DNA-templated, circadian rhythm, circadian regulation of gene expression |  |
| PBFT1_Binpacker_32_BINPACKER.4877.1 | Solute carrier family *25* (mitochondrial iron transporter), member *28* | *SLC25A28* | 1 | 0 | transport, membrane, integral component of membrane |  |
| PBFT1_Binpacker_25_BINPACKER.18564.1 | *CWF19*-like *1*, cell cycle control | *CWF19L1* | 1 | 0 | - |  |
| PBFT1_Shannon_41_Shannon_PBFT1_k41_c1_96_11420_1142 | ENSPFOP00000013980.2: PREDICTED: lymphatic vessel endothelial hyaluronic acid receptor 1-like | *LYVE1* | 1 | 0 | hyaluronic acid binding, integral component of membrane, cell adhesion, membrane |  |
| PBFT2_SOAP_21_scaffold6587 | Nucleoporin *54* | *NUP54* | 1 | 0 | nuclear pore |  |
| PBFT3_OASES_41_Locus_39111_Transcript_1_1_Confidence_1.000_Length_1624 | Shwachman-Bodian-Diamond syndrome | *SBDS* | 1 | 0 | mature ribosome assembly, ribosome biogenesis, exocrine pancreas development, granulocyte differentiation, myeloid leukocyte differentiation |  |
| PBFT1_OASES_41_Locus_9969_Transcript_2_2_Confidence_0.750_Length_1526 | Nucleoporin *43* | *NUP43* | 1 | 0 | - |  |
| PBFT3_Binpacker_25_BINPACKER.1317.1 | ATP synthase, H+ transporting, mitochondrial F*1* complex, gamma polypeptide *1* | *ATP5C1* | 1 | 0 | proton-transporting ATP synthase complex, catalytic core F(1), ATP biosynthetic process, proton transport, ATP synthesis coupled proton transport, proton-transporting ATP synthase activity, rotational mechanism, ATP hydrolysis coupled cation transmembrane transport, ion transport, transport | Yes, aerobic metabolism |
| PBFT1_IDBA_71_transcript-71_9051 | EMSY *BRCA2*-interacting transcriptional repressor | *EMSY* | 1 | 0 | regulation of transcription, DNA-templated |  |
| PBFT1_Binpacker_25_BINPACKER.20297.1 | ENSTNIP00000014672.1: unnamed protein product | *-* | 1 | 0 | melanosome, melanosome transport |  |
| PBFT3_OASES_71_Locus_5859_Transcript_1_2_Confidence_0.750_Length_1611 | Fumarylacetoacetate hydrolase domain containing *1* | *FAHD1* | 1 | 0 | catalytic activity, metabolic process |  |
| PBFT1_Binpacker_25_BINPACKER.16891.1 | *SH3*-domain binding protein *2* | *SH3BP2* | 1 | 0 | positive regulation of signal transduction, signal transduction, SH3/SH2 adaptor activity |  |
| PBFT3_transabyss_31_J1145841 | DAZ associated protein *2* | *DAZAP2* | 1 | 0 | - |  |
| PBFT1_Binpacker_25_BINPACKER.7308.1 | Solute carrier family *4* (anion exchanger), member *1a* (Diego blood group) | *SLC4A1A* | 1 | 0 | ion transport, transport, membrane, integral component of membrane, inorganic anion exchanger activity, anion transmembrane transporter activity, anion transport, inorganic anion transport, anion transmembrane transport, erythrocyte differentiation, embryonic hemopoiesis |  |
| PBFT1_Binpacker_25_BINPACKER.9343.1 | ENSGACP00000019119.1: PREDICTED: pleckstrin homology domain-containing family O member 2-like isoform X2 | *PLEKHO2* | 1 | 0 |  |  |
| PBFT2_Shannon_31_Shannon_PBFT2_k31_c1_28_19890_1989 | ENSONIP00000006077.1: PREDICTED: insulin receptor substrate 1-B isoform X2 | *IRS1* | 1 | 0 | insulin receptor binding |  |
| PBFT1_SOAP_71_scaffold3807 | Transmembrane protein *59* | *TMEM59* | 1 | 0 | membrane, integral component of membrane |  |
| PBFT1_IDBA_71_transcript-71_4137 | ENSORLP00000003898.1: uncharacterized protein LOC101169137 isoform X1 | *-* | 1 | 0 | - |  |
| PBFT1_Binpacker_25_BINPACKER.14508.1 | ENSPFOP00000004932.2: PREDICTED: TIR domain-containing adapter molecule 1-like | *TICAM1* | 1 | 0 | innate immune response, signal transduction, signal transducer activity |  |
| PBFT1_Binpacker_32_BINPACKER.10953.1 | Stomatin (*EPB72*)-like *2* | *STOML2* | 1 | 0 | membrane |  |
| PBFT1_transabyss_51_R752365 | Hydroxyacyl-CoA dehydrogenase/*3*-ketoacyl-CoA thiolase/enoyl-CoA hydratase (trifunctional protein), beta subunit | *HADHB* | 1 | 0 | transferase activity, transferring acyl groups other than amino-acyl groups, transferase activity, transferring acyl groups, catalytic activity, metabolic process, transferase activity | Yes, β-oxidation |
| PBFT2_Binpacker_32_BINPACKER.1269.2 | ENSONIP00000003225.1: PREDICTED: TSC22 domain family protein 2 isoform X2 | *TSC22D2* | 1 | 0 | transcription factor activity, sequence-specific DNA binding, regulation of transcription, DNA-templated |  |
| PBFT2_transabyss_71_R378686 | Vinculin | *VCL* | 1 | 0 | actin filament binding, cell adhesion, actin binding, structural molecule activity, actin cytoskeleton |  |
| PBFT1_Binpacker_32_BINPACKER.2475.4 | Solute carrier organic anion transporter family, member *2*B*1* | *SLCO2B1* | 1 | 0 | ion transport, transport, transmembrane transport, transporter activity, plasma membrane, membrane, integral component of membrane |  |
| PBFT1_IDBA_71_transcript-71_21096 | Rho GTPase activating protein *29*b | *ARHGAP29B* | 1 | 0 | signal transduction, intracellular signal transduction, metal ion binding, intracellular, positive regulation of GTPase activity, GTPase activator activity |  |
| PBFT2_OASES_51_Locus_37529_Transcript_1_2_Confidence_0.500_Length_1780 | HtrA serine peptidase *1*b | *HTRA1B* | 1 | 0 | serine-type endopeptidase activity, regulation of cell growth, insulin-like growth factor binding, proteolysis, extracellular region |  |
| PBFT1_IDBA_71_transcript-71_36663 | Glycerol-*3*-phosphate dehydrogenase *1*c | *GPD1C* | 1 | 0 | oxidoreductase activity, oxidation-reduction process, protein homodimerization activity, carbohydrate metabolic process, oxidoreductase activity, acting on the CH-OH group of donors, NAD or NADP as acceptor, glycerol-3-phosphate metabolic process, NAD binding, glycerol-3-phosphate dehydrogenase [NAD+] activity, glycerol-3-phosphate catabolic process, glycerol-3-phosphate dehydrogenase complex | Yes, GO term enriched |
| PBFT2_OASES_31_Locus_8170_Transcript_10_10_Confidence_0.576_Length_1946 | Titin-cap | *TCAP* | 1 | 0 | Z disc, heart contraction, muscle cell development, T-tubule organization |  |
| PBFT2_SOAP_31_scaffold18533 | Unannotated | *-* | 1 | 0 | - |  |

Supplementary table 4. Gene ontology terms significantly enriched in the bluefin PhyloGWAS test

| Gene Ontology class | Gene Ontology term enriched | topGO ‘weight01’ algorithm fishers test *p* value |
| --- | --- | --- |
| Cellular Component | Glycerol-3-phosphate dehydrogenase complex | 0.00017 |
|  | Sarcoplasmic reticulum | 0.00017 |
| Molecular Function | Glycerol-3-phoshpate dehydrogenase [NAD+] activity | 0.00007 |
|  | NAD binding | 0.00087 |
| Biological Process | glycerol-3-phosphate catabolic process | 0.00011 |

**
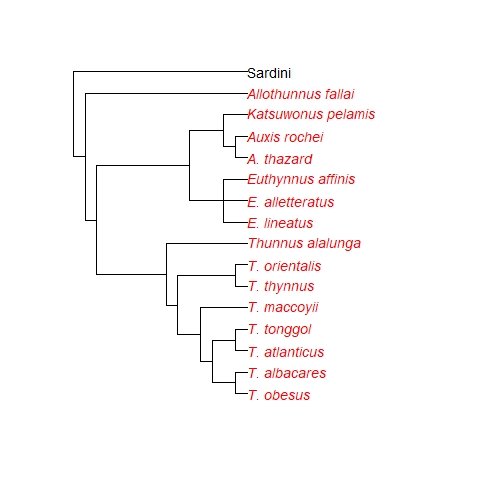
**

**Figure S1.** Hypothesised phylogenetic relationships amongst modern day tunas and their nearest ectothermic relatives, the Sardini. Redrawn from (Qiu et al. 2014; Díaz-Arce et al. 2016; Bernal et al. 2017). Endothermic species are highlighted in red.


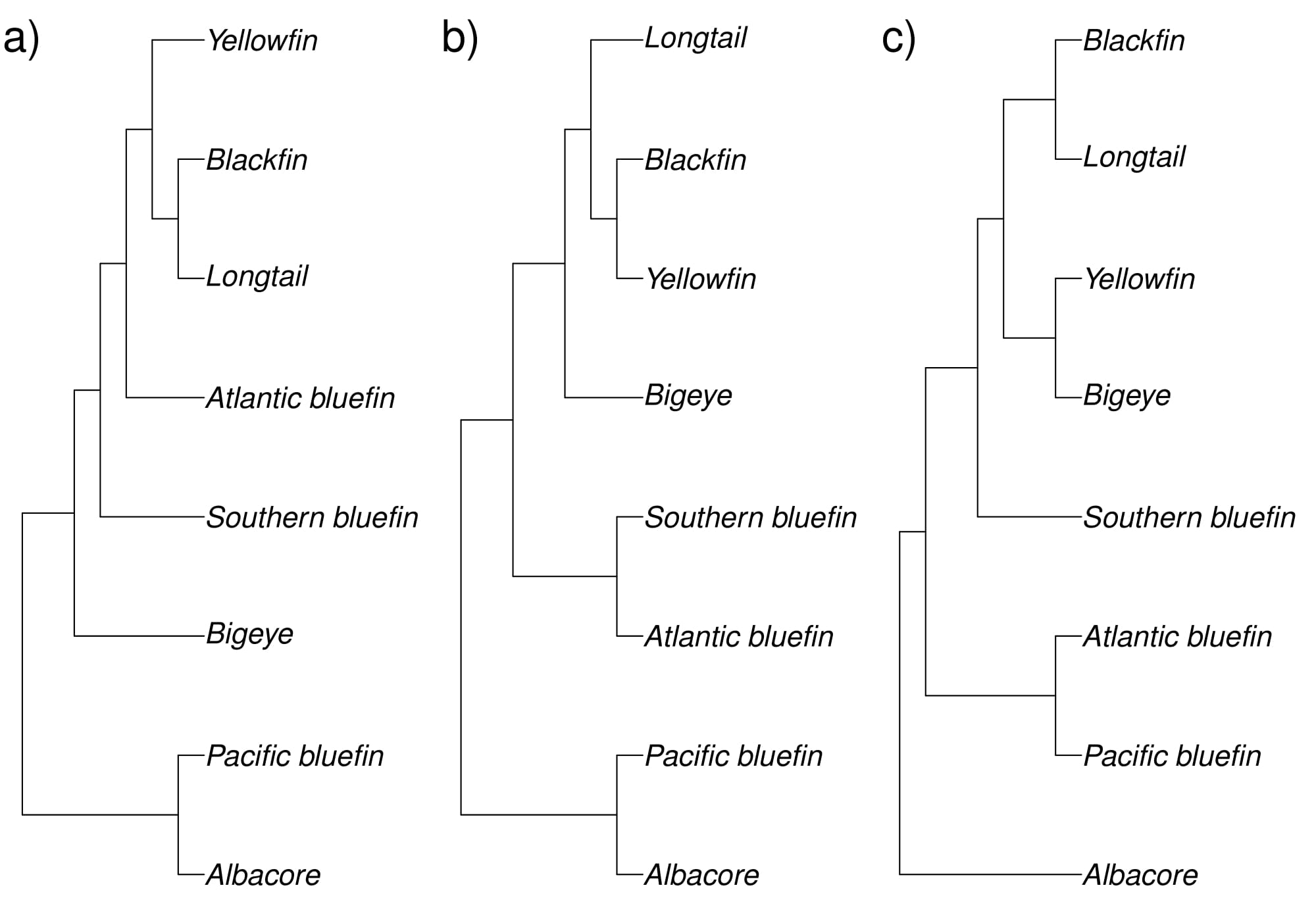


**Figure S2.** Previously published phylogenetic trees for the Thunnus tuna

.

a) uses mitochondrial cytochrome *b*, and first showed the mitochondrial sister relationship between Pacific bluefin and albacore tuna (Chow and Kishino 1995); b) is a recent complete mitochondrial genome maximum-likelihood tree (Bayona-Vásquez et al. 2017); c) is a partial genome-data RAD-seq maximum-likelihood phylogenetic tree, showing the paraphyly of bluefin tuna (Díaz-Arce et al. 2016)


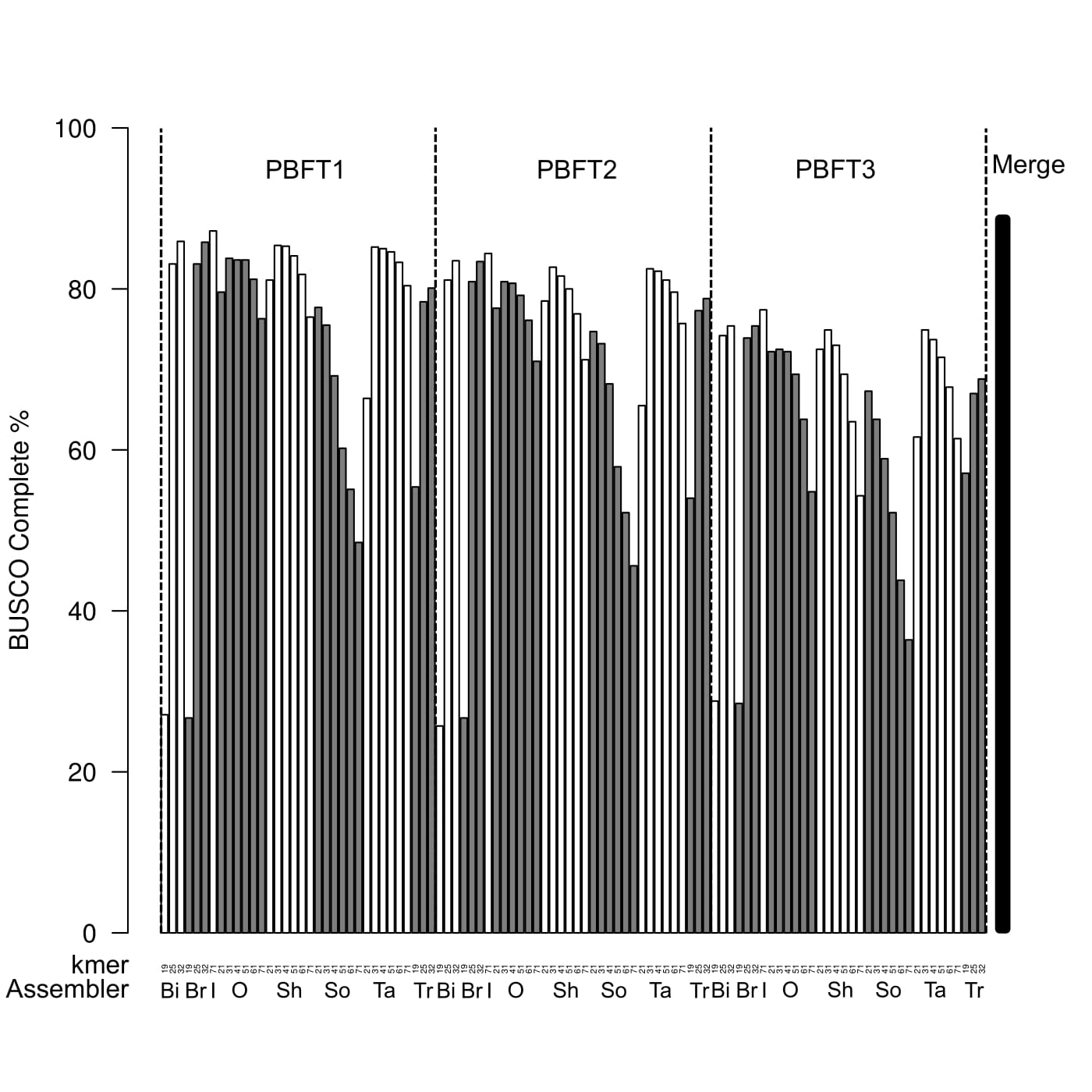


**Figure S3.** BUSCO scores (completedness %) for each of the individual assemblies (white and grey) and the overall merged assembly (thick and highlighted in black). Number on the first row of the x label refers to the kmer setting for the individual assembly. Abbreviation on the second row refers to the assembly software (Bi – Binpacker; Br – Bridger; O – Velvet-Oases; Sh – Shannon; SO – SOAPdenovo-trans; Ta – Trans-ABySS; Tr – Trinity. Assemblies from each individual Pacific bluefin (PBFT1, PBFT2, PBFT3) separated by vertical dashed line.

**
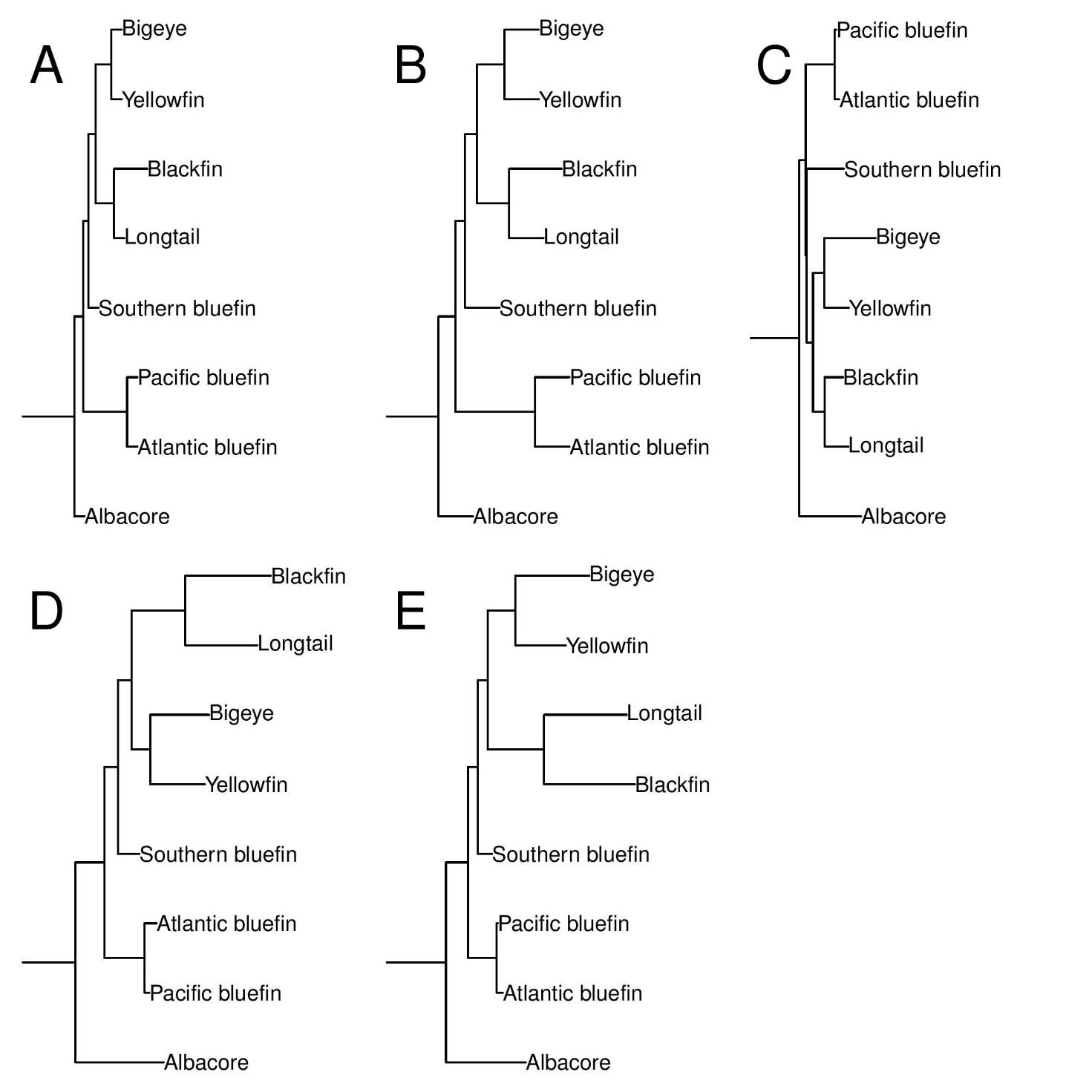
**

**Figure S4.** Phylogenetic trees reconstructed by different methods in this study. The top row shows phylogenies reproduced by multi-species coalescent (MSC) methods, the bottom row by supermatrix methods. a) shows a tree inferred by ASTRAL, where poorly supported (SH-like <10) nodes in input gene-trees are collapsed to hard polytomies; b) shows a tree inferred by ASTRAL where poorly supported gene trees are not collapsed; c) shows a tree inferred by SNaQ; d) shows a tree inferred by RAxML using concatenated transcripts; e) shows a tree inferred by ExaBayes using concatenated 4-fold synonymous sites. Arbitrary tip lengths were added to the two ASTRAL figures. Branch lengths in a, b and c are in coalescent units (number of generations/ effective population size). Branch lengths in d and e are in expected substitutions per site. All nodes are fully supported by local posterior probability =1 (a, b), bootstrap = 100% (d), or posterior probability = 1 (e). See materials and methods for details.


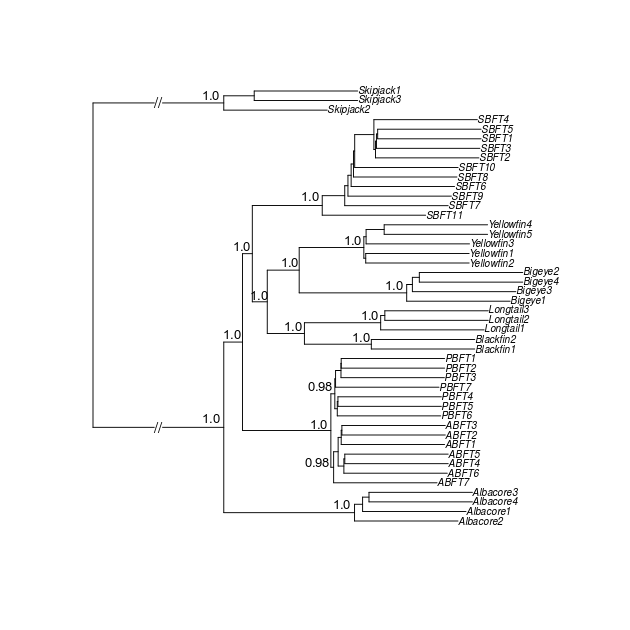


**Figure S5**. ASTRAL-inferred phylogenetic tree with monophyly of individuals not restrained, showing monophyly of each species. Local posterior probability is given for each node representing clades up to the species level.


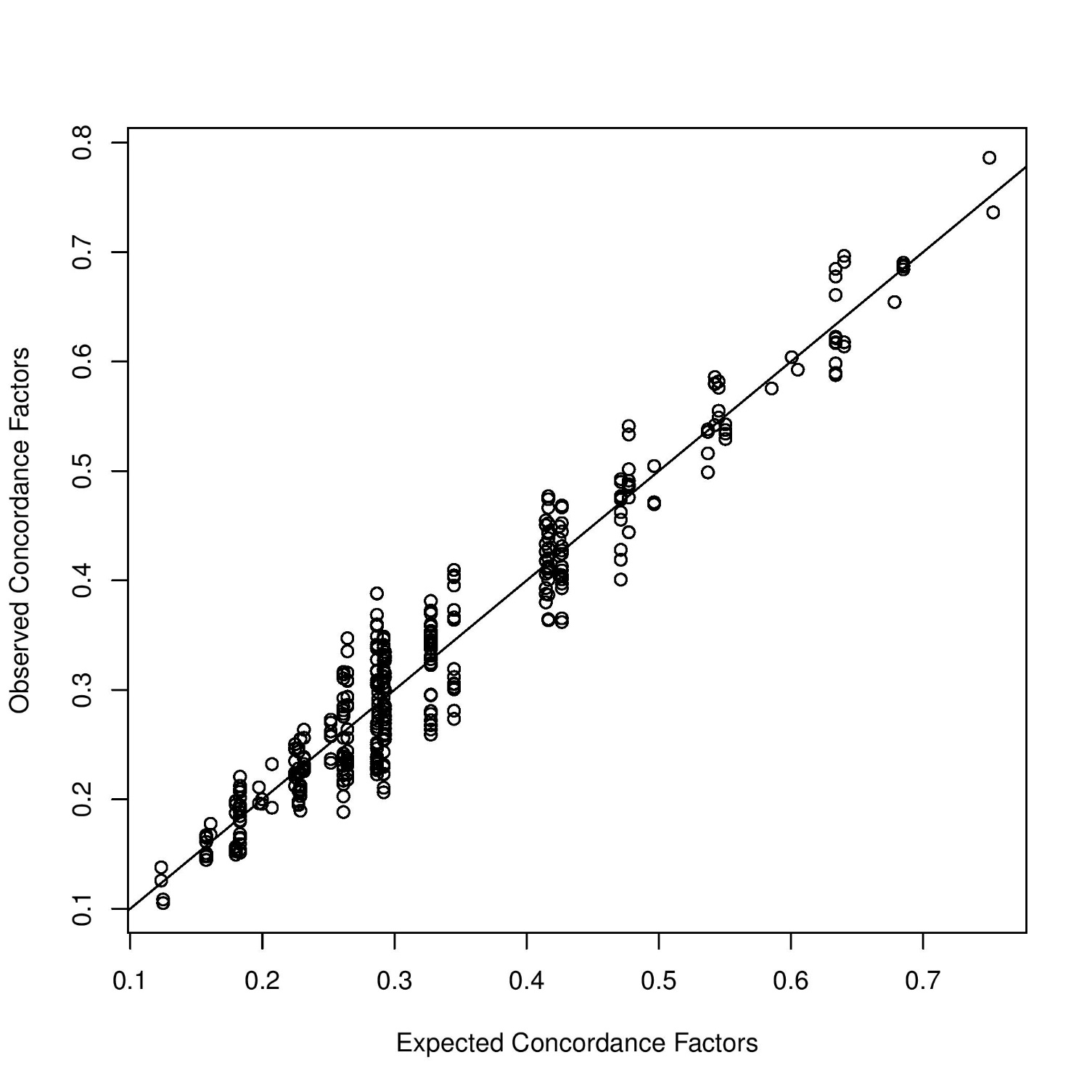


**Figure S6.** Observed concordance factors between tip quartet sets in gene tree dataset, estimated in “PhyloNetworks” julia packaged, plotted against expected concordance factors calculated in R package “phylolm” on phylogenetic tree inferred in PhyloNetworks under coalescence.


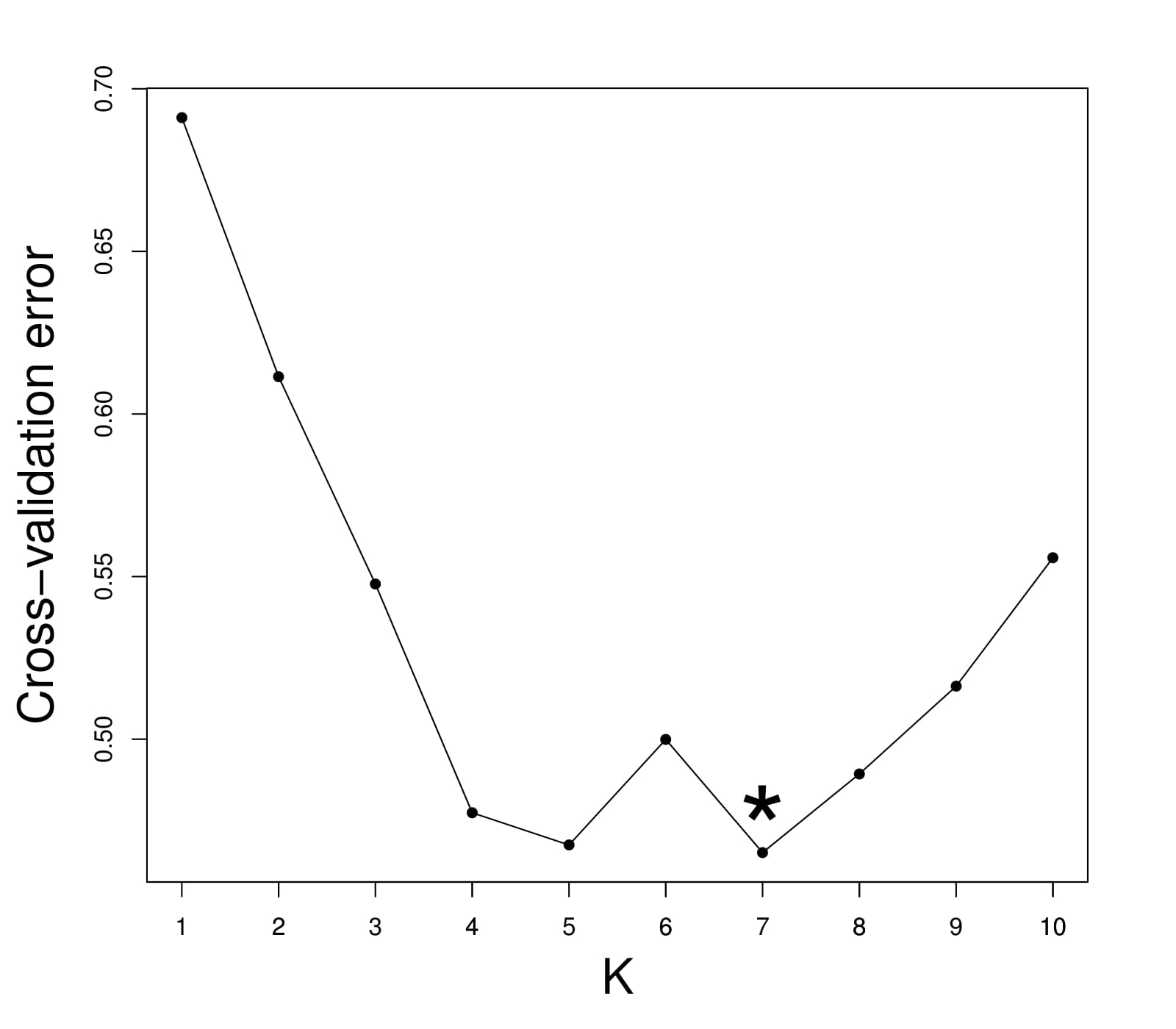


**Figure S7*.*** Cross-validation error scores for runs of ADMIXTURE on the *Thunnus* data set with different numbers of populations (*K*). The optimal score (*K*=7) is indicated with a *.


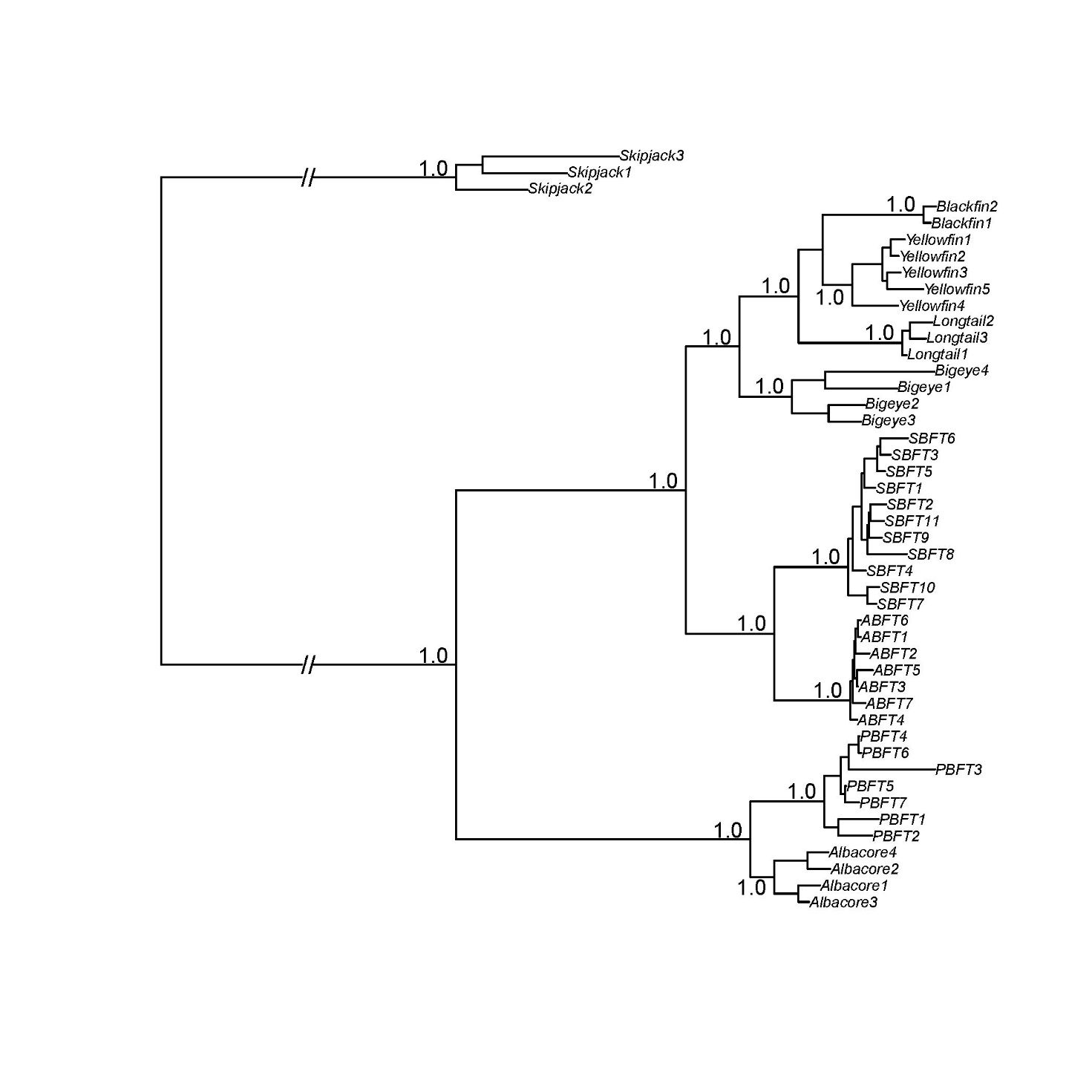


**Figure S8.** Phylogenetic tree inferred from a concatenation of the 13 mitochondrial genome genes. Posterior probability is given for each node representing clades up to the species level.

**
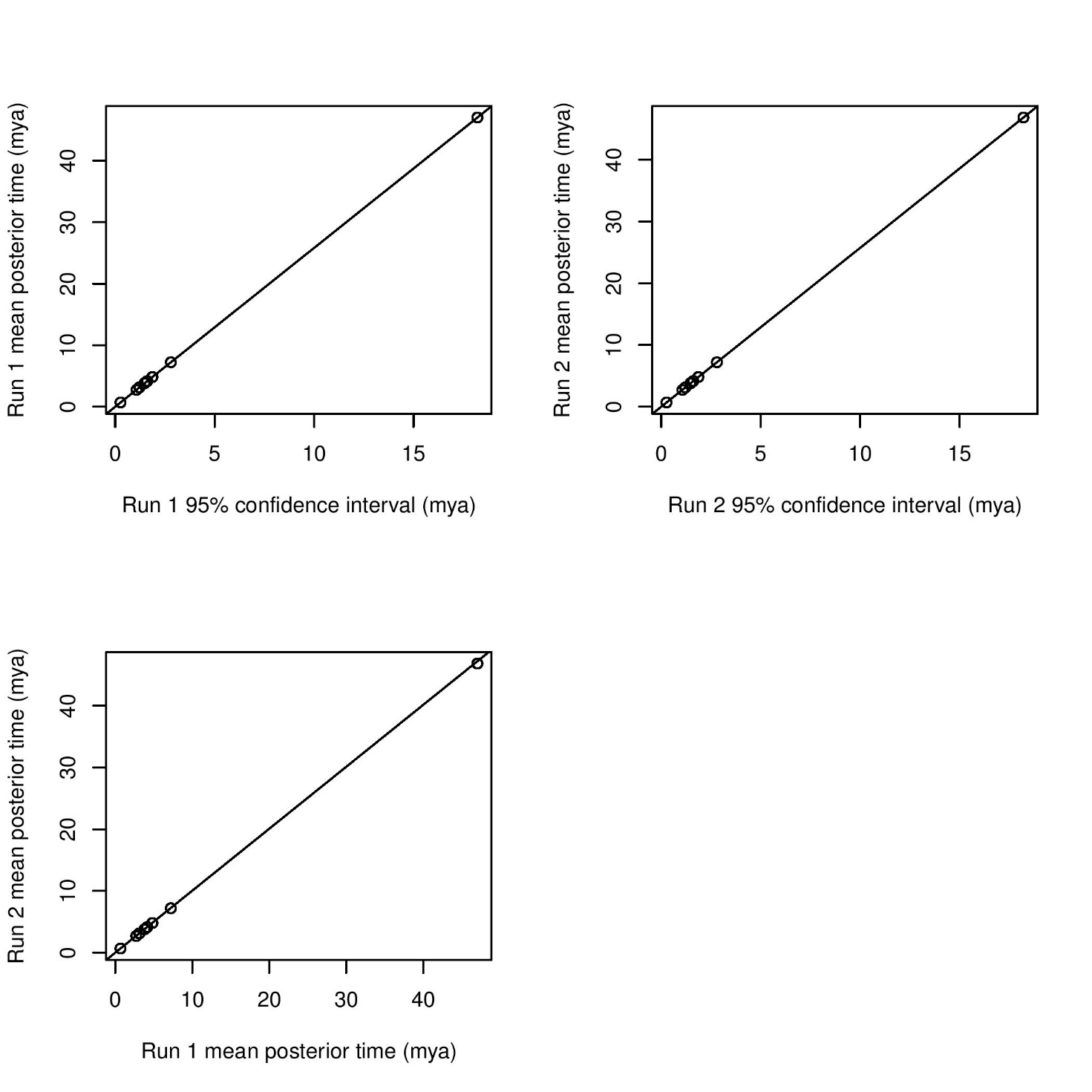
**

**Figure S9**. Panel a): infinite-site plots for MCMCTree run 1 (left) and run 2 (right), showing 95% confidence intervals increase linearly with estimated divergence mean dates. Panel b); MCMCtree mean posterior divergence times of run 1 (x axis) and run 2 (y axis) are almost identical.
